# From juvenile to adult: investigating miRNAs, gene expression, and the juvenile cone in olive development

**DOI:** 10.3389/fpls.2025.1682101

**Published:** 2025-10-29

**Authors:** Paola Romero-Rodríguez, Ana Gordon, Esteban Meca, Carmen Tercero-Alcázar, Galen T. Martin, M. Teresa Garcia-Lopez, Juan Moral, Brandon S. Gaut, Concepción M. Diez

**Affiliations:** ^1^ Department of Agronomy, University of Córdoba, Córdoba, Spain; ^2^ Department of Cellular Biology, Physiology and Immunology, University of Córdoba, Córdoba, Spain; ^3^ Department of Applied Physics, Radiology and Physical Medicine, University of Córdoba, Córdoba, Spain; ^4^ Department of Ecology and Evolutionary Biology, University of California, Irvine, Irvine, CA, United States

**Keywords:** juvenile-to-adult transition, microRNAs, qPCR, high throughput sequencing, methylome, selection markers, breeding

## Abstract

The long juvenile phase in perennials hinders rapid breeding, highlighting the need for early selection markers. Some species, such as the olive tree (*Olea europaea* L.), develop a juvenile cone, where adult tissue forms in the upper and peripheral canopy, while basal and inner regions remain juvenile. These structures offer a unique, yet underexplored, system for studying the juvenile-to-adult transition while minimizing genetic and environmental variability. We analyzed tissues from trees with a juvenile cone to identify genes and miRNAs distinguishing juvenile from adult vegetative tissue. Known transition markers, including miR156, miR172, and homologs of *APETALA2* and *AGAMOUS-like 42*, showed clear differential expression across developmental stages, with the miR156/miR172 ratio being particularly discriminatory. In contrast, DNA methylation patterns showed few differences between juvenile and adult leaves, and differentially expressed genes were not enriched for methylation changes. Our findings show that the juvenile cone represents an intermediate developmental stage and provides a unique system for studying phase transitions in perennials. Identified miRNA and gene markers not only improve our understanding of olive development but also offer practical tools to facilitate the selection of rapid-maturing genotypes in olives and other perennials, such as pistachio (*Pistacia vera* L.). Overall, the juvenile cone serves as a valuable model for developmental analyses, and our findings provide a framework to enhance breeding efficiency in olives and other perennial fruit species.

## 1 Introduction

Plants undergo distinct developmental phases during their life cycles, including embryogenesis, juvenile vegetative growth, adult vegetative growth, and eventually, reproduction (Huijser and Schmid, 2011). In a physiological context, the juvenile phase (JP) consists of plants that form true leaves and axillary buds but are not capable of flowering and reproduction. Plants transition from JP into an adult vegetative phase to eventually become reproductively competent (Huijser and Schmid, 2011). In many plants, including common models like *Arabidopsis* and less-studied species like eucalyptus (*Eucalyptus globulus*), vegetative tissues differ morphologically between juvenile and adult phases, a phenomenon known as heteroblasty (Wang et al., 2011; Poethig, 2013). The heteroblastic transition from juvenile to adult tissue occurs within weeks or months after germination in annual plants, but this transition can take many years in perennial plants (Gaut et al., 2015). The long duration of JP in these crops hinders the ability of breeders to rapidly develop new varieties to meet market demands and to adapt to shifting environmental pressures. Despite the fact that the farmgate value of perennial fruit and nut trees is substantial – i.e., more than half that of annual cereals ($451B vs. $837B of US dollar/year) (Schreinemachers et al., 2018), the juvenile-to-adult transition remains poorly studied in perennial crops.

The juvenile-to-adult transition has, however, been studied previously by contrasting juvenile and adult vegetative tissues (Teotia and Tang, 2015). Although there are still open questions, it is clear that miRNA-mediated regulation is an important contributor to this heteroblastic transition (Wang et al., 2009; Huijser and Schmid, 2011; Lian et al., 2021). Plant miRNAs regulate gene expression post-transcriptionally by binding to the complementary sequence of a specific target mRNA, initiating a cascade that leads to mRNA degradation and/or translational repression of targeted genes (Reinhart et al., 2002). Two miRNA families have been found consistently to be involved in the juvenile-to-adult transition: miR156 and miR172 (Poethig, 2013; Zhao et al., 2022). The miR156 family is a master regulator of juvenility that is highly expressed in seedlings but decreases towards the adult phase (Chuck et al., 2007; Wang et al., 2009; Zheng et al., 2019; Raihan et al., 2021). Across diverse species like maize and *Arabidopsis*, miR156 targets *SQUAMOSA PROMOTER BINDING PROTEIN-LIKE* (*SPL*) transcription factors, which in turn influence a diverse set of developmental processes (Zheng et al., 2019; Vander Schoor et al., 2022). In contrast to miR156, the miR172 family is lowly expressed in juveniles but highly expressed in adult tissue. miR172 represses *APETALA2* (*AP2*)-like genes, key regulators of both flowering and the juvenile-to-adult transition (Wollmann et al., 2010; Vander Schoor et al., 2022). Finally, it is also worth noting that both miR156 and miR172 are multi-copy, at least in *Arabidopsis* (Reinhart et al., 2002; Jung et al., 2007; Teotia and Tang, 2015). Although the presence of multiple copies likely ensures functional redundancy, individual family members can vary in their spatial, temporal, and environmental expression patterns (Yang et al., 2013; Lian et al., 2021).

In perennial species, the juvenile-to-adult transition can take a specific form that is called a “cone of juvenility” or a juvenile cone (Hartmann et al., 2002). This structure presents a spatial disposition in which the upper and peripheral canopy are mature, while the basal and inner regions remain juvenile (Hackett, 1985). As a result, juvenile tissues are localized within a cone-shaped area comprising the trunk and the bases of the lower branches (Hackett, 1985; Hartmann et al., 2002). Thus, the juvenile cone phase represents a potential intermediate between a completely juvenile plant and a fully reproducing adult. In some heteroblastic perennial trees the juvenile cone phase, is visually recognizable. This characteristic presents a unique opportunity to study developmental differences between juvenile and adult tissue in a single plant (or genotype) at a single point in time. It is worth noting that juvenile cones can be present across a diverse array of perennial species, including *Malus* spp., *Citrus* spp., silver birch (*Betula verrucosa* Ehrh.), the honey locust (*Gleditsia triacanthos* L.), the European beech (*Fagus sylvatica* L.) (Hackett, 1985) and olives (*Olea europaea* L.). To our knowledge, however, juvenile cones have been largely ignored by developmental biologists and molecular biologists, although they have been used in the context of plant propagation (Hartmann et al., 2002). Juvenile cones may represent an ideal system to study the genetic and epigenetic mechanisms that govern the juvenile-to-adult transition in plants, but expression patterns between their juvenile and adult tissues has never been evaluated or compared with true juvenile (seedling) and fully adult trees.

Our study focuses on juvenile cones in olives, the most extensively planted fruit crop globally (FAOSTAT, 2025). Our study has two overarching goals. The first is identifying genes and miRNAs that correlate with differences between juvenile and adult vegetative tissues. Are the same miRNAs and target genes implicated in olive as for other plant species? There has been little research on this question in perennials generally and in olives specifically. However, one gene called *JAT* (the *JUVENILE-TO-ADULT TRANSITION* gene) has been identified as a regulator of olive juvenile-to-adult transition (Fernández-Ocaña et al., 2010), but little is known about gene function, regulation, or even its underlying genetic sequence. The second goal is applied, which is to progress toward identifying molecular markers that characterize developmental phases. In theory, these markers can ultimately be deployed to manipulate olive genetically or to predict the duration of JP of individual genotypes. In olive breeding, JP is currently shortened by forcing seedlings growth under optimal conditions (Santos-Antunes et al., 2005; Rallo et al., 2018), successfully reducing its duration from ~ 15 years to 3–4 years in some cases (Rallo et al., 2018). At present, plant height is the marker utilized for JP duration, but it is inefficient. Plant height allows olive breeders to discard ~40% of seedlings with predictably long JPs (De La Rosa et al., 2006; Rallo et al., 2008) but fewer than 50% of the remaining plants flower within four years (De La Rosa et al., 2006). These results highlight the need to develop molecular markers that are more efficient for the early selection of olives and that could perhaps be applied to other fruit crops to increase the efficiency of breeding perennial species.

**Figure 1:**
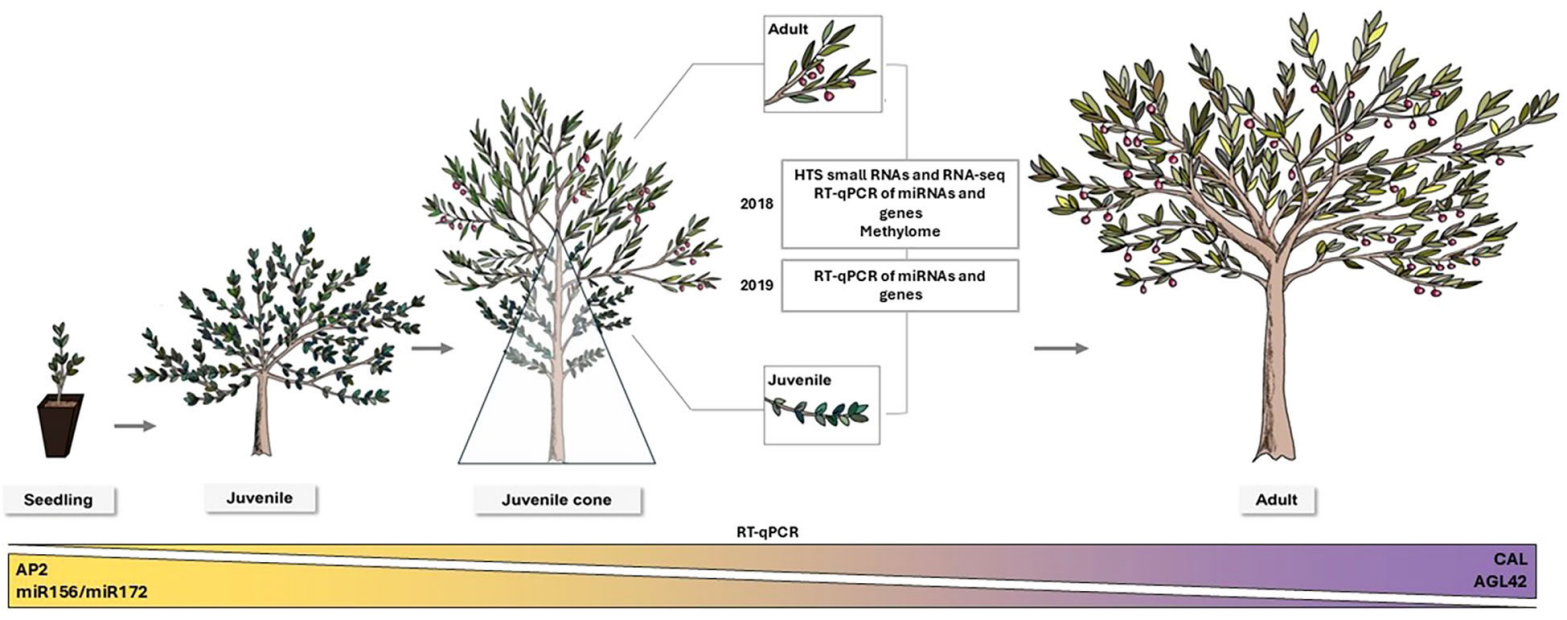
A schematic of juvenile-to-adult development in *Olea europaea* L. Seedlings emerging from germination (left) exhibit a deeply juvenile morphology and during the first years (up to 10–15 years in natural conditions). Trees remain juvenile until the transition to adult phase starts. This transition takes the form of a recognizable juvenile cone (middle). The triangle depicts the juvenile cone, where the bottom and closer branches to the trunk remain juvenile while the upper part of the canopy is adult. The insets indicate leaves and branches in juvenile and adult phases on the same tree that were sampled to conduct this study. Eventually, the plants transition to becoming fully adult. A schematic overview of the experiments conducted is included. At the bottom of the figure, expression patterns validated by RT-qPCR for the miRNA ratio miR156/miR172 and the candidate genes *AP2*, *AGL42*, and *CAL* during tree development are displayed.

To study the juvenile-to-adult transition in olives, we have generated high-throughput sequencing (HTS) libraries of gene and miRNA expression data from juvenile and adult leaf tissues from trees with a juvenile cone. By comparing expression between these two developmental stages, we identify differentially expressed miRNAs and genes. We further verify their expression by studying expression across a wider range of developmental stages, including early seedlings, fully juvenile plants, and fully adult (reproductive) plants (**Figure 1**). Finally, we hypothesize that there may be epigenetic triggers to the juvenile-to-adult transition since epigenetic regulation can play an essential role in the control of gene expression during plant development (Bitonti et al., 2002). As a first step, we have investigated differential methylation patterns between juvenile and adult leaves, a contrast that has not been studied in olives or (to our knowledge) in any other plant species. Our results highlight the role of the juvenile cone as unique system to study the juvenile-to-adult transition in plants and open potential avenues for the development of marker-assisted-selection to shorten the JP and accelerate breeding in olive and other perennials.

## 2 Results

### 2.1 Differential expression between juvenile cone and adult cone tissue

We generated two sets of high throughput sequence (HTS) data, focused on either miRNA or gene expression. Both sets were generated from the same seven olive trees. Of these, five were 5-year-old full siblings with juvenile cones; we sampled both juvenile and adult leaves from them. We refer to these leaves as “juvenile cone” and “adult cone” tissue (**Figure 1**, **Supplementary Figure S1**). We also included leaves from a fully adult and a fully juvenile tree.

#### 2.1.1 Differentially expressed miRNAs

We constructed 16 small RNA libraries representing 12 distinct samples of juvenile and adult leaves, along with additional replicates. After HTS sequencing of each library, we identified 2,928,984 different small RNA sequences in total. Of the ~3M small RNAs, 1,186,745 (or 40.5%) mapped against non-coding transcripts from the reference annotation. The remaining non-mapping sequences may correspond to mRNA fragments or may originate from non-coding transcripts that have not been annotated. After the removal of tRNA fragments, the remaining 1,134,164 were distinct, expressed small RNA sequences, of which 640,239 (56.5%) corresponded to ribosomal RNA (rRNA) fragments. We performed differential expression analysis by contrasting all juvenile samples (that is juvenile cone tissue and tissue from the juvenile plant; *n* = 6 trees) to all adult samples (adult cone + adult; *n* = 6 trees), both excluding and including rRNA sequences. Without rRNA sequences, a single differentially expressed miRNA was identified: oeu_miR156_1 (FDR ≤ 0.006), which was classified as a miR156 based on perfect matches in miRBase (**Table 1**) (Kozomara et al., 2019). The inclusion of rRNA fragments in the analyses increased the common dispersion parameters and thereby increased the number of differentially expressed sequences to eight (FDR ≤ 0.01). One was oeu_miR156_2, and the remaining seven were identified as isomiRNAs of oeu_miR172 (**Table 1**) via miRbase. The two miRNAs exhibited expression patterns consistent with previous studies (Wu et al., 2009; Wang et al., 2011; Raihan et al., 2021), in that oeu_miR156_2 was more highly expressed in juvenile tissue, with a log fold change (logFC) of 5.06 relative to adult tissue. In contrast, oeu_miR172 was more highly expressed in adult tissue, with a logFC equal to –2.56 in juvenile versus adult tissue. Thus, our work uncovered the two canonical miRNA families that have been implicated in aging in other species (Wang et al., 2011).

**Table 1:** Olive mRNAs differentially expressed between juvenile and adult cones, based on high-throughput sequencing (HTS) data, and their mature sequences.

miRNA name^1^	Sequence-5p (5’- 3’)	*p*-value	η^2^
oeu_miR156_1	UGACAGAAGAGAGUGAGCAU	0.0009791	0.53754
oeu_miR156_2	UGACAGAAGAGAGUGAGCAG	0.0003336	0.62540
oeu_miR159	UUUGGAUUGAAGGGAGCUCUA	0.0157600	0.30457
oeu_miR172^2^	AGAAUCCUGAUGAUGCUGCAU	0.0002644	0.64426

1. Sequences assigned to miRNA families using miRbase.

2. This sequence represents the primary miR172 miRNA identified by Differentially expressed genes (DEG) analyses of HTS data, but six other miR172-related sequences were also identified under less stringent analysis conditions. Results of Kruskal-Wallis test on expression data, as measured across five developmental stages, based on miRNAs.

We also examined a third subfamily, oeu_miR159, because previous studies have implicated miR159 in targeting *MYB* transcription factors that affect gibberellin response pathways, flowering time and anther development (Millar and Gubler, 2005; Schwab et al., 2005). We identified a highly expressed miR159 (oeu_miR159, see **Table 1**) as well as another highly expressed member of the miR156 family (denoted oeu_miR156_1 in **Table 1**) that were differentially expressed but not significantly so, with FDRs of 0.18 and 0.12 respectively. Nonetheless, we chose to include them in downstream experiments (see below) based on the previous literature, high expression, and notable logFC differences between juvenile and adult tissue (logFC of 1.48 for oeu_miR156_1 and 10.91 for oeu_miR159).

#### 2.1.2 Differentially expressed genes

**Figure 2:**
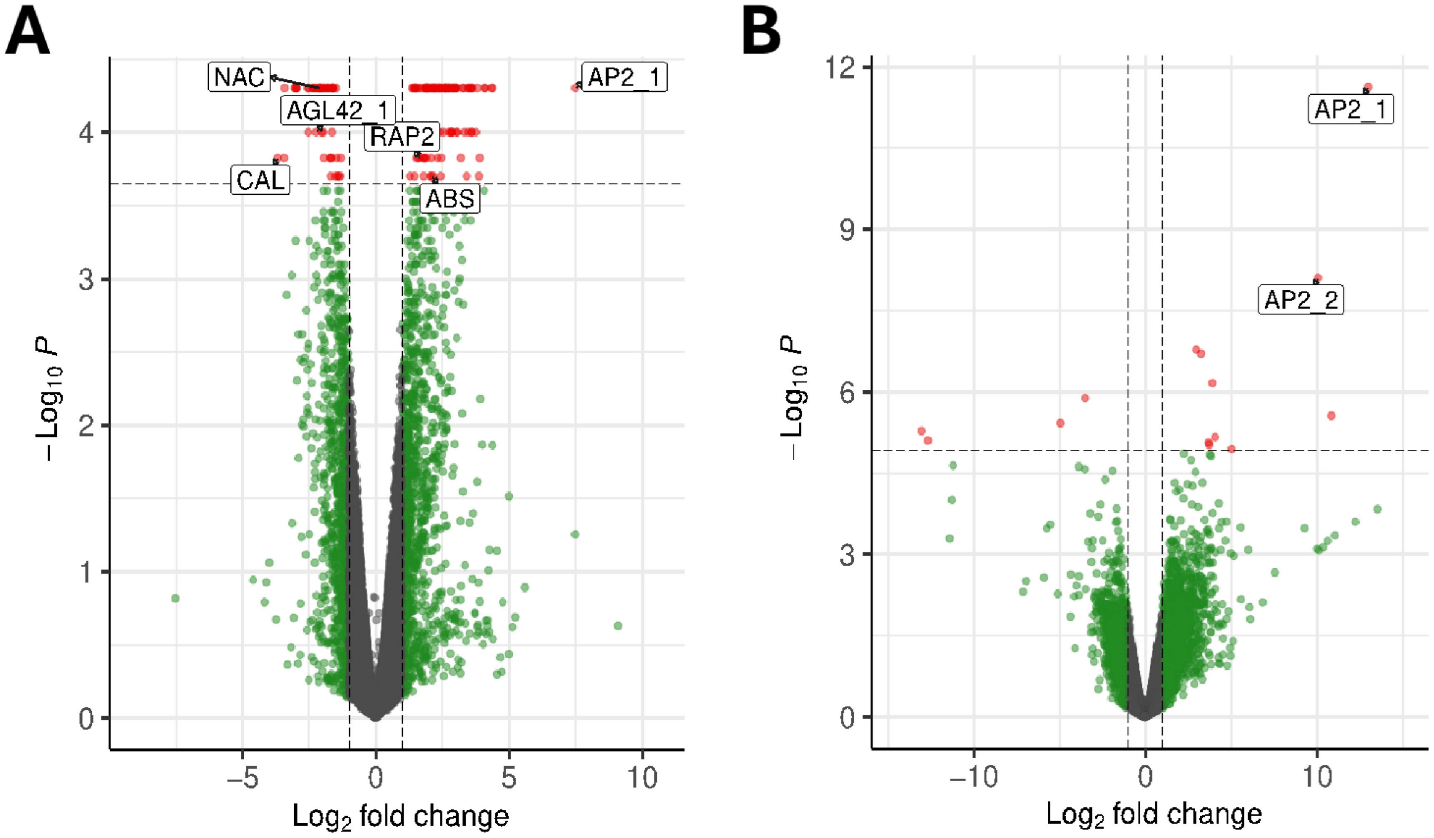
Analyzing differential gene expression between juvenile and adult leaves. **(A)** A volcano plot based on CuffDiff analyses, plotting the log10 fold-change (x-axis) against log P-value (y-axis). The dotted horizontal line represents the significance of q-valued cutoff of P ≤ 0.0001. The labels represent some of the candidate genes chosen by potential function in developmental phase transitions. **(B)** A similar diagram based on EdgeR analyses, with the dotted horizontal line representing FDR ≤ 0.00001. The APETALA2_1 (AP2_1) and APETALA_2 (AP2_2) paralogs were two of the four genes found by both analysis methods.

We also evaluated differentially expressed genes (DEGs) by generating RNA-seq libraries from the same 16 tissue samples. We evaluated DEGs between adult and juvenile samples using two alternative algorithms: EdgeR and Cuffdiff (**Figure 2**). One analytical approach, based on EdgeR identified 17 DEGs between juvenile and adult tissue. A second, based on CuffDiff, identified 169 DEGs, four of which were also identified by EdgeR (**Supplementary Table S1**). Out of the total of 182 DEGs, 35% (or 64 genes) were unannotated, reflecting the dearth of functional information in olives. We further refined the list of candidate genes to provide a more tractable sample for further RT-qPCR analyses. To filter the 182 candidates, we considered the significance value (p-value and FDR) of each DEG and evaluated their potential role in the juvenile-to-adult transition based on GO annotations (Ashburner et al., 2000; Aleksander et al., 2023). For each gene, we sought evidence for a role in developmental, flowering, or hormonal functions, reasoning that these functions are likely to be involved in the developmental transition or act as suitable markers of that transition (**Table 2**). We ultimately narrowed our candidate set to nine genes, including two *AP2* homologs (**Figure 2**), which we named *AP2_1* and *AP2_2*; two genes potentially involved in abscisic stress ripening; a gene similar to a MADS-box gene; two genes related to *AGAMOUS-like 42* (*AGL42*), a central regulator of floral meristem identity; and a transcription factor with homology to the *CAULIFLOWER* locus (*CAL*). We also included a gene with an unknown function (*UNK*) that had the highest significance level from CuffDiff analyses (**Table 2**). It is worth mentioning that the *JAT* gene, which was previously identified as regulator of the juvenile-to adult transition in olive (Fernández-Ocaña et al., 2010), was not annotated in the Farga v6 olive reference genome (Oev6). Since the gene sequence is unavailable, we were unable to confirm that *JAT* is differentially expressed.

**Table 2:** Candidate genes assayed with RT-qPCR chosen based on differential expression (EdgeR and/or CuffDiff) from high-throughput sequencing (HTS) data, with putative functions.

Gene name	Putative function	Gene ID	logFC^1^	EdgeR^2^	CuffDiff^2^
*ABS*	Abscisic stress ripening	OE6A069433	2.107	–	0.049
*AP2_1*	*AP2* ERF and B3 domain-containing transcription factor	OE6A019660	13.031	8.19E-08	0.020
*AP2_2*	*AP2* ERF and B3 domain-containing transcription factor	OE6A026361	10.100	1.82E-04	–
*CAL*	truncated transcription factor CAULIFLOWER A-like	OE6A018260	-3.657	–	0.041
*AGL42_1*	MADS-box AGL42-like isoform X1	OE6A063960	-2.227	–	0.031
*AGL42_2*	MADS-box AGL42-like isoform X2	OE6A063960	-2.227	–	0.031
*RAP2*	ethylene-responsive transcription factor RAP2-7-like isoform X2	OE6A012609	1.665	–	0.041
*NAC*	NAC domain-containing 35-like	OE6A105042	-2.091	–	0.020
*UNK*	Unknown	OE6A058044	16.710	2.97E-76	0.049

1. Log fold-change between juvenile and adult tissue, with positive values indicating higher expression in juvenile tissue.

2. Two different algorithms were used for the calculation of the differential expression, CuffDiff (q-Value) and EdgeR (FDR).

### 2.2 RT-qPCR analyses of candidate miRNAs and genes

#### 2.2.1 miRNA expression ratios varied by developmental stage

Given the differentially expressed miRNAs identified with HTS data, we performed RT-qPCR of these miRNAs on two sets of trees. The first was a set of five trees with juvenile cones that were also used to generate HTS data; these trees were sampled in two years (2018 and 2019). The second set encompassed carefully staged trees in distinct seedling, juvenile, juvenile cone, adult cone, and adult phases; at least five trees were represented in each category for all assays (**Supplementary Table S2**).

**Figure 3:**
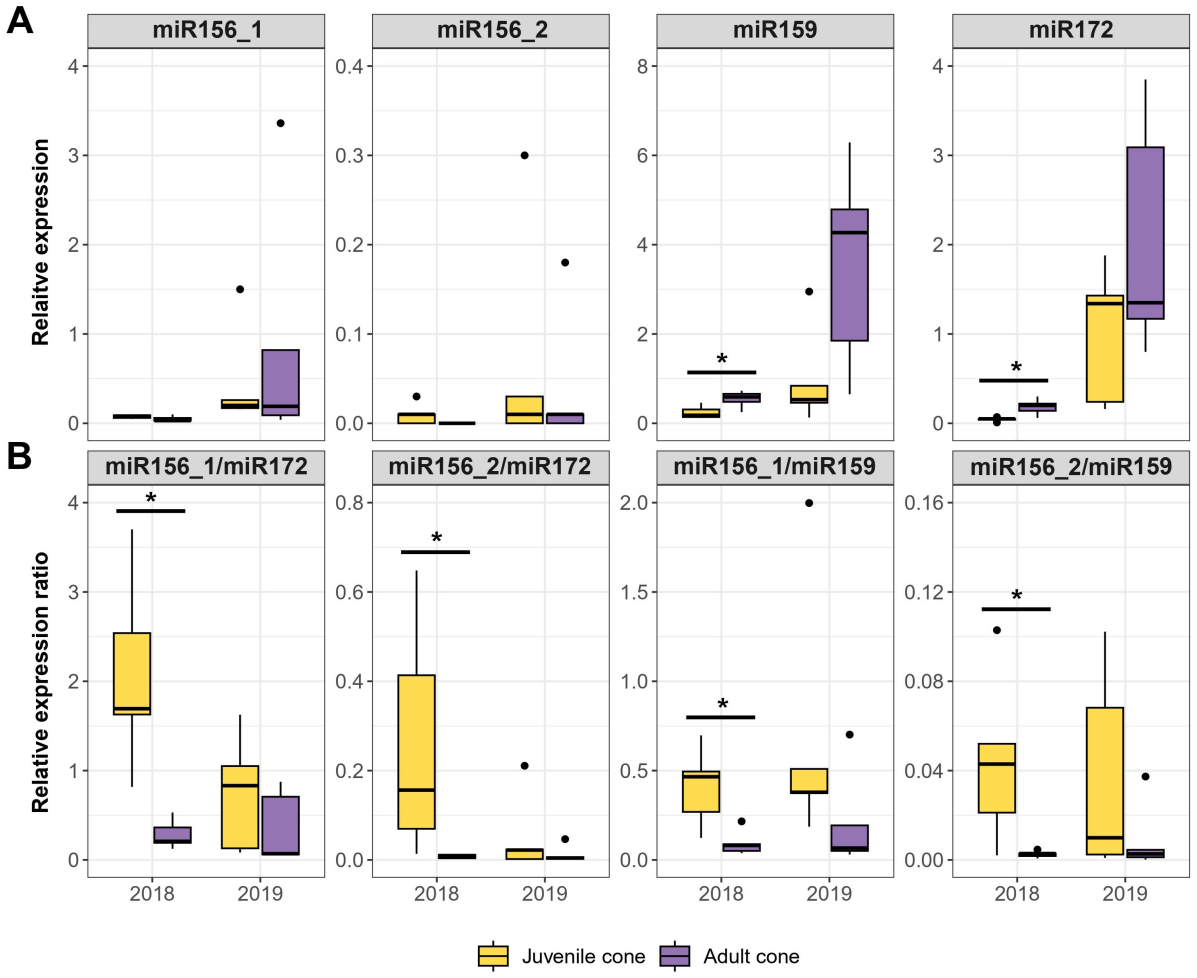
RT-qPCR based on four candidate miRNAs in two years. **(A)** Expression relative to a reference (y-axis) for each miRNA in juvenile cone and adult cone tissue over consecutive years (2018 and 2019). The assays were based on the same five olive trees in both years. **(B)** The relative expression ratios for two miRNAs typically provided more consistent contrasts than individual miRNAs. In each panel, the boxplots represent the range of values between Q1 (25%) and Q3 (75%). The black line in the boxplot represents the median (Q2). Dots outside the whiskers represent outliers. Significant differences between groups were calculated by Wilcoxon Rank test (P ≤ 0.05). The different groups were determined using Dunn’s test (P ≤ 0.05). Significant differences between groups are indicated: *P ≤ 0.05 (Wilcoxon Rank test).

We first measured the expression of the four candidate miRNAs in the original five trees with juvenile cones, contrasting juvenile to adult tissue in two years (2018 and 2019). For these analyses, we used 5SRNA for RT-qPCR normalization, following previous studies (Lin and Lai, 2013; Song et al., 2016). Normalized miRNA expression for both tissue stages was generally higher across tissues in 2019 than in 2018, and the RT-qPCR results between development stages were not always consistent with HTS-based inferences. For example, the HTS data indicated higher expression of miR156_1 and miR156_2 in juvenile tissue, which was not always obvious from the RT-qPCR data (**Figure 3A**). Similarly, miR172 expression did not follow the expected higher expression in adult vs. juvenile tissue; indeed, in one year, expression was higher in the juvenile tissue (**Figure 3A**). However, building on the suggestion of Wu et al. (2009) that there may be a feedback loop between miR156 and miR172, we hypothesized that the ratio of miRNAs could be more informative than either marker alone. Indeed, the miR156/miR172 ratio was consistently higher for juvenile compared to adult tissue in both years, although only significantly different in 2018 (**Figure 3B**). We believe the better consistency of the ratio reflects a balance of the two markers. miR156 promotes juvenility, while miR172 is associated with the induction of adult traits, so that a high miR156/miR172 ratio indicates a juvenile state, while a lower ratio signals progression toward adulthood. Similar ratios calculated with miR159, as opposed to miR172, were also consistent across tissues and years (**Figure 3B**, **Supplementary Figure S2**).

**Figure 4:**
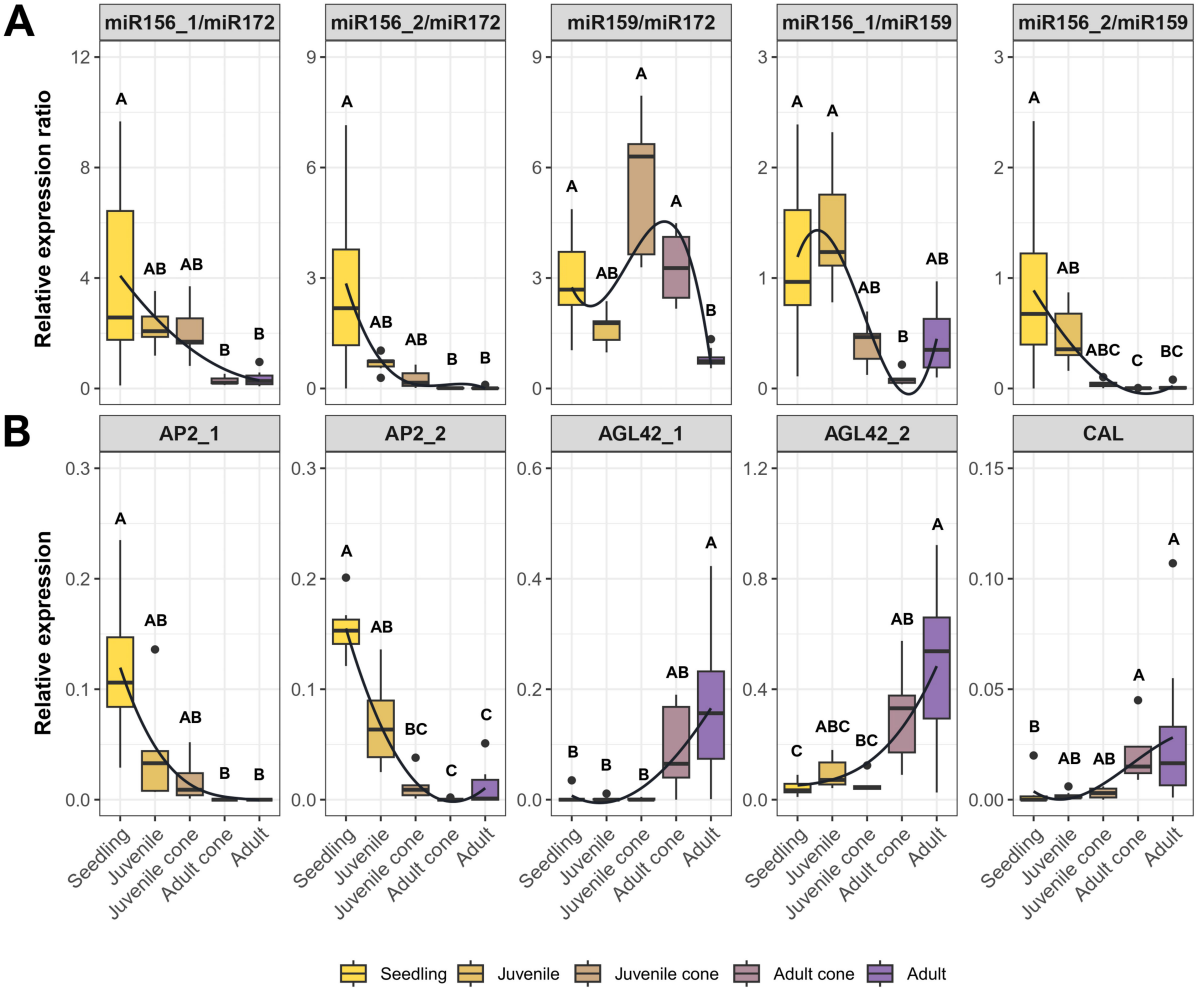
RT-qPCR results based on miRNA ratios and candidate genes across five tissue types. **(A)** Expression ratios based on the four candidate miRNAs in **Table 1**. **(B)** Expression of five of the candidate genes listed in **Table 2**. Each plot represents RT-qPCR relative expression in five tissue types - Seedling, Juvenile, Juvenile Cone, Adult Cone, and Adult. Each box plot represents the range of values between Q1 (25%) and Q3 (75%). The black line in the boxplot represents the median (Q2). Dots outside the whiskers represent outliers. The curves were fitted to show trends, based on a polynomial function. Significant differences between groups were calculated by Kruskal-Wallis test (P < 0.05). The different groups, signified by letters above the boxplots, were determined using Dunn’s test (P < 0.05).

To further test the idea that miRNA ratios may be useful markers of JP, we performed RT-qPCR in a larger set of trees representing five stages, from seedling to fully adult (**Figure 1**). We analyzed the data in two ways. First, we tested whether developmental stage was a significant factor explaining miRNA expression (Kruskal-Wallis; P ≤ 0.01). The Bonferroni-corrected results were significant for three of four individual miRNAs and all miRNA ratios (**Tables 1**, **S3**). The developmental stage explained a substantial proportion of expression variance– i.e., η^2^ values ranged from 0.30 for miR159 to as high as 0.74 for the miR159/miR172 ratio (**Tables 1**, **S3**). Second, we qualitatively examined expression patterns, hoping to find markers that yielded a consistent gradient across developmental stages. The individual miRNAs were not useful in this context (**Supplementary Figure S3**), but three ratio measures were promising. The ratios miR156_1/miR172, miR156_2/miR172, and miR156_2/miR159 all exhibited gradual declines from seedling to adult tissue (**Figure 4A**).

#### 2.2.2 Candidate gene expression across the juvenile-to-adult transition

We identified nine candidate genes from HTS data (**Table 2**) and investigated their expression using RT-qPCR across developmental stages. Of the nine, we validated the expression of seven (*AP2_1*, *AP2_2*, *AGL42_1*, *AGL42_2*, *CAL*, *NAC*, and *UNK*). Five of these seven differed in expression between juvenile and adult leaves (Wilcoxon test; P ≤ 0.05) in 2018, based on data from the five plants with juvenile cones. Among the five genes, *AP2_1* and *AP2_2* had higher expression in juvenile cone tissue, but *AGL42_1*, *AGL42_2*, and *CAL* exhibited higher expression in adult leaves (**Supplementary Figure S4**). Expression of these five genes (**Figure 4B**), measured in at least five samples per stage (**Supplementary Table S2**), confirmed that developmental stage was a significant factor (P ≤ 0.001; Kruskal-Wallis). Moreover, developmental stage explained substantial proportion of the variance in gene expression (η^2^ > 0.46 for all genes; **Supplementary Table S3**). Note, however, that we were unable to detect expression of some genes in some tissues. For example, *AP2_1* expression was not detected in adult cone or adult tissue, while *AGL42_1* was not expression in most seedling, juvenile, and juvenile cone samples.

### 2.3 Relationships between miRNA targets and candidate genes

Candidate miRNAs are expected to have sequence homologies to specific target genes. For example, miR172 targets *AP2* in *Arabidopsis* and other species, which explains negative expression correlations between *AP2* and miR172 in those species (Aukerman and Sakai, 2003; Yant et al., 2010). We assessed potential target genes for candidate miRNAs based on BLAST sequence identity analyses. We followed the previous convention by considering ‘hits’ (i.e., potential targets) as genes with ≤ 3 mismatches in the reverse (3’ to 5’) direction. For example, oeu_miR172 yielded 15 BLASTn hits that overlapped annotated genes in the *O. europaea* L. Ensemblplants database (https://plants.ensembl.org/; last accessed Dec 9, 2024). Of these 15, seven were in the reverse orientation expected for miRNA targeting. Six of the seven genes had homology to floral homeotic *APETALA 2-*like isoforms, based on UniProt predictions. We note, however, that miR172 hit neither *AP2_1* (OE6019660) nor *AP2_2* (OE6026361) because there were > 3 mismatches. Thus, we cannot be confident that oeu_miR172 is the direct regulator of *AP2_1* and *AP2_2* genes. Nonetheless, these sequence relationships provide strong evidence that the oeu_miR172 targets genes in the *AP2* gene family.

We performed similar analyses for miR156_1 and miR159. For the latter (miR159), we detected three genic overlaps in reverse orientation; two were homologous to *GAMYB* transcription factors. MiR156_1 had 19 gene hits with 13 in the reverse direction. As expected, 12 of these had homology to *SQUAMOSA PROMOTER BINDING PROTEIN-*like genes, confirming *SPL* genes are likely targets of oeu_miR156. The remaining gene was an *AP2* homolog (OE6032696), which suggests an interesting structure/function relationships between miR156 and *AP2* genes. In *O. europaea* L., one miR156 homolog is located physically between the *AP2_1* and *AP2_2* genes, about ~20kb from either gene. Another miR156 is located directly upstream from the *AP2_1* gene (**Supplementary Figure S5**), with highly correlated expression to *AP2_1* across the HTS samples (R^2^ = 0.89; Pearson).

### 2.4 Methylome analyses

#### 2.4.1 Genome wide DNA-methylation

**Figure 5:**
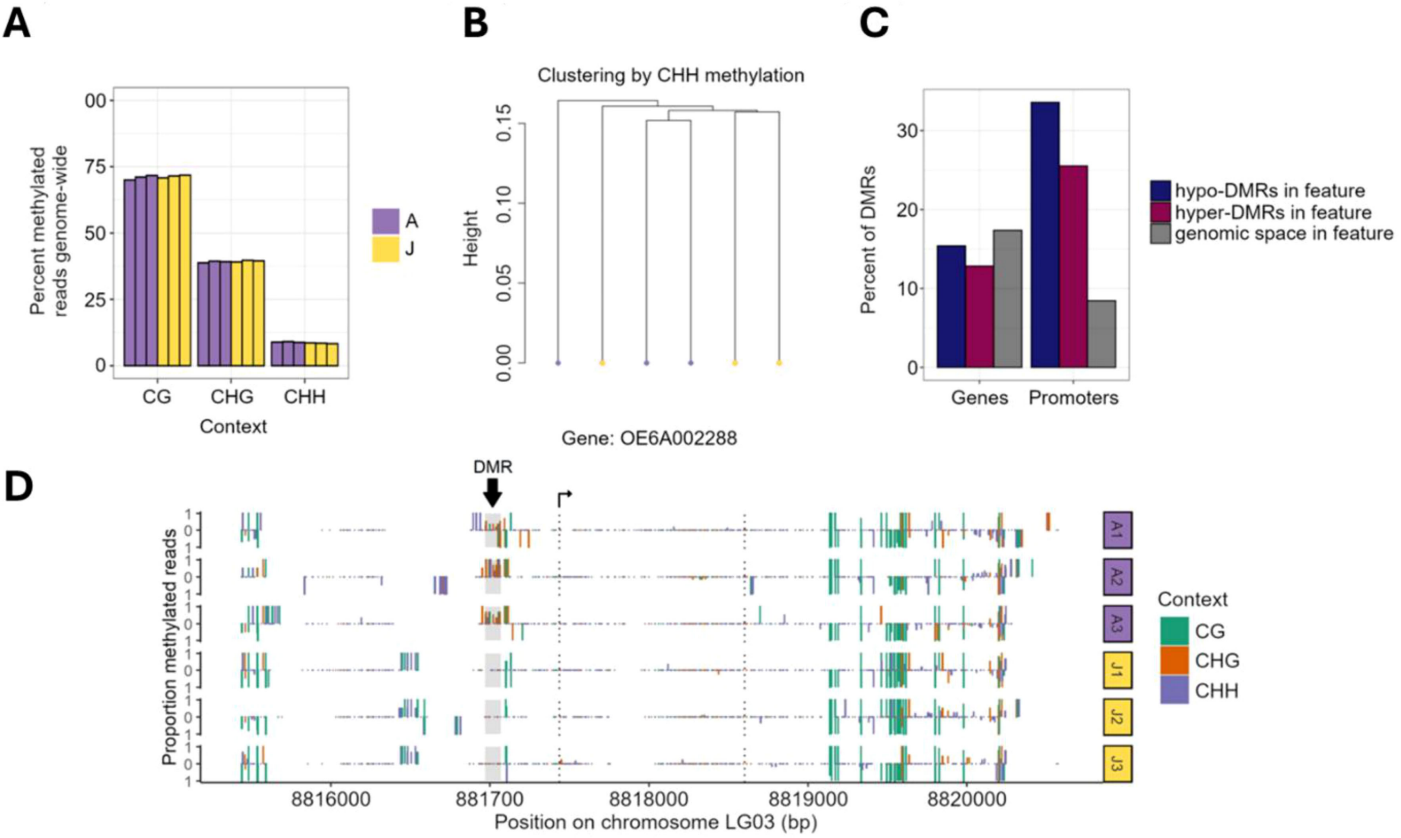
Analysis of methylation data of juvenile cone and adult cone tissue of olive trees. **(A)** Plots of the percentage of methylated reads in the three cytosine contexts in adult (A) and juvenile (J) cone tissues. The bars represent results from each of the three biological replicates for each tissue type. **(B)** A dendrogram clustering six samples by the shared CHH methylation status of individual nucleotides based on genome-wide data. The adult and juvenile tissues do not form monophyletic groups. **(C)** Analysis of the differentially methylated regions (DMRs) shows that genes have a similar proportion of DMRs. Hyper-methylated DMRs represent regions methylated in adult cone but not juvenile cone tissue, with hypo-methylated DMRs exhibiting the opposite pattern. Only 13.0% of hyper and 16.1% of hypomethylated DMRs were found in genes, representing a significant underrepresentation under random expectations based on genomic space. **(D)** An example of one gene (OE6A002288) with a DMR between juvenile cone and adult cone tissue. Most DMRs are only in the CHH context, but this example has evidence for differentiation in all three methylation contexts (CG, CHG and CHH). A1, A2, and A3 refer to the three adult replicates, with J1, J2, and J3 referring to juvenile replicates.

Considering that epigenetic phenomena can contribute to developmental transitions (Brink, 1962; Fraga et al., 2002; Xu et al., 2018), we investigated the epigenetic state of juvenile cone and adult cone tissues, using DNA methylation. Since our work represents the first characterization of the olive methylome, we began by characterizing genome-wide methylation patterns. The patterns were generally similar to other angiosperms (Niederhuth et al., 2016). Methylation was highest in methylated CG cytosines (mCG), where the genome-wide weighted mCG level was 70.8%; intermediate in mCHG (39.1%); and low in mCHH (8.6%). These patterns did not differ significantly between adult and juvenile cone tissue (**Figure 5A**) – e.g. mCG levels were 71.46% in the juvenile cone vs 70.93% in the adult cone (P > 0.01; Student’s T-test) with similar results for mCHG (39.43% vs 39.13%) and mCHH (8.47% vs 8.93%). Like other angiosperms, CG methylation was elevated within genes (**Supplementary Figure S6**) and higher in all three contexts (CG, CHG and CHH) for transposable elements (**Supplementary Figure S7**). Finally, we also clustered samples based on individual base calls. They did not clearly group together by tissue type in the CG (**Figure 5B**) and CHG contexts (**Supplementary Figure S6**), again suggesting little epigenetic differentiation between tissues. Juvenile and adult cone tissue did cluster separately based on CHH methylation (**Supplementary Figure S7**), but overall, there was little signal of distinct methylation patterns between juvenile cone and adult cone tissue.

#### 2.4.2 Local differentiation

We also characterized regions of local differentiation between juvenile cone and adult cone samples by examining 100 bp sliding windows across the genome, using MethylKit (Akalin et al., 2012). We tested for significant deviations between the adult and juvenile samples in each window and each sequence context separately. For ease of presentation, we defined “hypermethylated” differentially methylated regions (DMRs) as windows with higher methylation levels in the adult cone phase, and “hypomethylated” with higher methylation in juvenile cones. In total, we identified 743 mCHH DMRs (619 hypermethylated and 124 hypomethylated), 13 mCHG DMRs (5 hypermethylated and 8 hypomethylated), and 18 mCG DMRs (7 hypermethylated and 11 hypomethylated). These DMRs represent a tiny fraction (i.e., ~5.95 x 10-5 %) of the ~1.34 Gb genome assembly and were significantly underrepresented in genic space (chi-square test, P ≤ 0.001, χ^2^ = 8.00) (**Figure 5C**).

We also investigated promoter regions, defined as the 2.0 kb region upstream of the transcription start site (TSS). Unlike in genes, DMRs were overrepresented in promotor regions (chi-square test, P ≤ 2.2e-16, χ^2^ = 242.74), with 161 (25.5%) of hypermethylated and 48 (33.6%) hypomethylated DMRs found within 8.44% of genomic space (**Figure 5C**). Genes with DMRs in their gene body or promoter were not enriched for any specific function based on gene ontology analysis. Furthermore, the set of DEGs from RNAseq analyses had fewer DMRs (either hypo or hypermethylated) than expected compared to the complete gene set. In fact, only a few of the DEG sets contained DMRs: four genes more highly expressed in adult cones had mCHH DMRs (OE6A103168 and OE6A101780 were hypomethylated DMRs and OE6A072380 and OE6A008998 were hypermethylated) and two genes that were under-expressed in adults contained mCHG hypermethylated DMRs (OE6A063960 and OE6A084474) (**Figure 5D**). We also examined the candidate genes (**Table 2**) to assess whether they contained DMRs, but none did. Overall, there was little evidence to suggest that DNA methylation differences correlate with, or contribute to, differential genic expression between juvenile and adult cone tissue.

## 3 Discussion

Juvenile cones have been characterized in perennial species for decades (Hackett, 1985), but few studies have taken advantage of this structure to characterize developmental processes. Here, we have used juvenile and adult tissues from trees with an evident juvenile cone to characterize molecular markers of the juvenile-to-adult transition. By contrasting genetically identical tissue, our overarching goal has been to understand the molecular causes and correlations of this transition in olives. This information can be applied to accelerate breeding in olives and perhaps other perennial crops (Wang et al., 2011). In this study, we: i) identified miRNAs and genes that differ in expression between juvenile cone and adult cone tissue; ii) assessed the expression of these candidates in an expanded breadth of tissues at different stages of maturity; iii) showed that miRNA ratios reflect a progression of development stages; and iv) explored the potential for underlying epigenetic shifts correlated with tissue differences. Our results are unique, because they contrast heteroblastic tissues from trees with juvenile cones. In the process we have uncovered miRNAs and genes that act as markers of juvenility and may be useful for perennial breeding. Our work also highlights the value of the juvenile cone as a developmental system to study the juvenile-to-adult transition in perennials, where life cycles are long and genomic resources are limited compared to annuals and model plants.

### 3.1 Candidate miRNAs and genes involved in the juvenile-to-adult transition in olives

We have identified well-known miRNAs as markers of the juvenile-to-adult transition. Our analyses of high-throughput sequencing (HTS) data detected miR172 and miR156 family members (oeu_miR156_1 and oeu_miR156_2) as significantly differentially expressed between juvenile cone and adult cone tissues. These observations extend previous work showing that miR156 and miR172 have functional consequences for the juvenile-to-adult transition. For example, constitutive expression of miR156a in *Arabidopsis* delays the transition to adult tissue, resulting in a late flowering phenotype (Wu et al., 2009); reduced miR156 expression triggers early flowering (Wu et al., 2009); and miR172 overexpression leads to accelerated flowering (Aukerman and Sakai, 2003; Lian et al., 2021). In addition, studies across a range of taxa – including perennial species like avocado (*Persea americana*), *Populus*, *Eucalyptus* and oaks (*Quercus* sp.) – support the idea that miR156 and miR172 synchronize the juvenile-to-adult transition (Wu et al., 2009; Wang et al., 2011). Our work is consistent with this mechanism also acting in olive. It is worth reiterating that miR156 and miR172 have antithetical expression patterns, with miR156 more highly expressed in juvenile leaves and miR172 more highly expressed in adult leaves (Wang et al., 2011).

We also studied miR159, which was reported to induce the juvenile-to-adult transition and to play a key role in regulating miR156 expression in *A. thaliana* (Guo et al., 2017). Guo et al. (2017) found that plants deficient for miR159 exhibited an extended JP and elevated levels of miR156 compared to normal plants. This dynamic may perhaps reflect the targeting and repression of *R2R3 MYB* domain transcription factors by miR159. In *Arabidopsis*, miR159 specifically targets *MYB33* and *MYB65*, which are responsible for promoting programmed cell death in anthers and the endosperm (Alonso-Peral et al., 2010). In olive, the HTS data indicate that miR159 has large fold change differences between juvenile and adult cone tissue. Further RT-qPCR assays established miR159 expression as a reasonable marker of juvenility with substantial variation across maturation stages (**Figure 3**, **Supplementary Figure S3**).

Our work also reinforces previous work about the expression dynamics of genes previously identified as central to developmental processes. Among the ~170 genes found to be differentially expressed based on HTS data, we focused on nine candidates based on their level of significance, their fold change between tissue types, and their putative functions. Two of these were *AP2* homologs, *AP2_1* and *AP2_2*. The function and evolution of *AP2* genes have been studied widely in numerous taxa. They are known to repress flowering in species such as *A. thaliana*, rice, and maize (Chuck et al., 2007; Yant et al., 2010) and are more highly expressed in juvenile tissue (Huijser and Schmid, 2011; Teotia and Tang, 2015). Our RT-qPCR assays uncovered the expected pattern of *AP2* expression; both homologs were highly expressed in juveniles but lowly expressed in adults, which is the countervailing pattern to miR172 (**Figure 4**). An interesting note about *AP2_1* (and to a lesser extent for *AP2_2*) is that it appears to represent a hard switch that is fully off in adult tissue, at least within the detection limits of our assays (**Figure 4**). This pattern is consistent with the conserved miR172–*AP2* regulatory module, in which miR172 accumulation promotes the juvenile-to-adult transition by suppressing *AP2* activity (Aukerman and Sakai, 2003; Teotia and Tang, 2015). *AP2* also affects the broader miR156–*SPL* network. In this network, declining miR156 levels enable *SPL* transcription factors to activate miR172, which reinforces the juvenile-to-adult switch (Wu et al., 2009; Yant et al., 2010).

Some of the other candidate genes – such as *AGL42_1*, *AGL42_2*, and *CAL* – increase in expression with age and tissue maturation (**Figure 4**). *AGL42* is a MADS-box gene closely related to *SOC1* and is an essential regulator in the floral transition in *A. thaliana* (Dorca-Fornell et al., 2011; Liu et al., 2020). *CAL* is a floral meristem identity gene homologous to the flowering integrator *APETALA 1* (*AP1*) (Ferrándiz et al., 2000). In species such as *A. thaliana*, *Gossypium hirsutum*, and *Juglans regia*, *CAL* coordinates flower development by participating in the specification of floral organs and the transition from the inflorescence meristem to the flower meristem (Ferrándiz et al., 2000; Hassankhah et al., 2020; Cheng et al., 2021). Our analyses suggest that they may perform similar functions in olive.

We did not detect differential expression of *SPL* genes with our HTS data, which is surprising because they are primary miR156 targets (Zheng et al., 2019; Vander Schoor et al., 2022). One possible explanation is that expression differences between juvenile cone and adult cone tissues is less pronounced between fully adult and fully juvenile tissue. However, this argument does not hold for genes like *AP2_1* and *AP2_2*, so we tend to discount this explanation. Another possibility is that annotation inaccuracies contribute to the lack of differential-expression signals for SPL genes. Finally, given that there are 151 SPL-like genes annotated in the olive reference genome, it is entirely possible that miR156 affects many genes but each of them only subtly.

### 3.2 Novel insights about the juvenile-to-adult transition, with potential applications to breeding

Our work has contributed to several novel insights. This is, for example, the first examination of juvenile transition in olive at the scale of gene and miRNA expression. This study is also the first to utilize juvenile cones in fruit crops to study the heteroblastic transition from juvenile to adult tissue. In the many perennial crops that have them, juvenile cones provide some distinct experimental advantages. One advantage is that they permit sampling of juvenile and adult tissue from the same tree at the same time, thus reducing (or eliminating) variance related to genotypes, time, and environment. This decrease in noise may explain, in part, why our HTS-based differential expression analyses were so precise – e.g., identifying only a few bona fide miRNA candidates and a reasonable number of differentially expressed genes (DEGs) that included well-known genes. We have also uncovered interesting properties of these tissues. For some of our biomarkers, juvenile cone and adult cone tissues represent an apparent continuum in a developmental gradient (**Figure 1**). For example, juvenile cone tissue is less pronounced for some miRNA ratios compared to leaves from seedlings and fully juvenile trees (**Figure 4**). Thus, trees with juvenile cones may represent an intermediate stage that, in some respects, is neither completely juvenile nor fully adult. These tissues help establish a gradient across stages of developmental maturity in olives (**Figure 1**) and may do so in other crops as well.

We have also performed DNA methylome analyses in olive, the first in this economically important species. Our analyses were motivated by the hypothesis that the juvenile-to-adult transition could be mediated by epigenetic shifts. However, we found little evidence to support this hypothesis on a genome-wide scale because there was almost no overall difference in methylation levels or patterns between juvenile cone and adult cone tissue. In retrospect, our results may be unsurprising, because previous methylome studies have generally found little intra-individual methylation variation between tissues (Roessler et al., 2016), except for specialized tissues such as endosperm and pollen vegetative nucleus (Lauria et al., 2004; Hsieh et al., 2009; Zemach et al., 2010). Nonetheless, our data indicate that olive has DNA methylation patterns that are generally typical of other angiosperms, in terms of the locations of methylation (genes vs. TEs; **Supplementary Figure S8**) and relative levels of methylation across cytosine contexts (**Figure 5A**). We note that olive does, however, have a relatively low percentage of gene-body methylated (gbM) genes (at 16.3%) compared to the angiosperm range (from ~10% to 61%) (Niederhuth et al., 2016). There is debate about the function of gbM, but it does correlate with gene expression levels and variances (Muyle and Gaut, 2018; Muyle et al., 2021, 2022).

There were also few differentially methylated regions (DMRs) between juvenile and adult tissue. The few DMRs were typically in the CHH context, likely reflecting active deposition of CHH methylation in differing tissues. Nonetheless, there are some puzzling aspects of these DMRs. One is that they are directional: significantly more were hyper-methylated in adult cone tissue compared to juvenile cone tissue (**Figure 5C**). Another is that they do not correlate with expression – e.g., DMRs were not over-represented within DEGs. It is still possible that a few of these DMRs contribute to functional shifts between tissue types. It is also possible that other chromatin and epigenetic changes affect the juvenile-to-adult transition. In Arabidopsis, for instance, the decrease in miR156 transcription, leading to vegetative phase change, correlates with an increase in the amount of the epigenetic mark H3K27me3, as well as an increase in H3K27ac modification in its promoter (Xu et al., 2016). A promising avenue for future work will be to investigate differentiation in additional chromatin markers between heteroblastic tissues.

**Figure 6:**
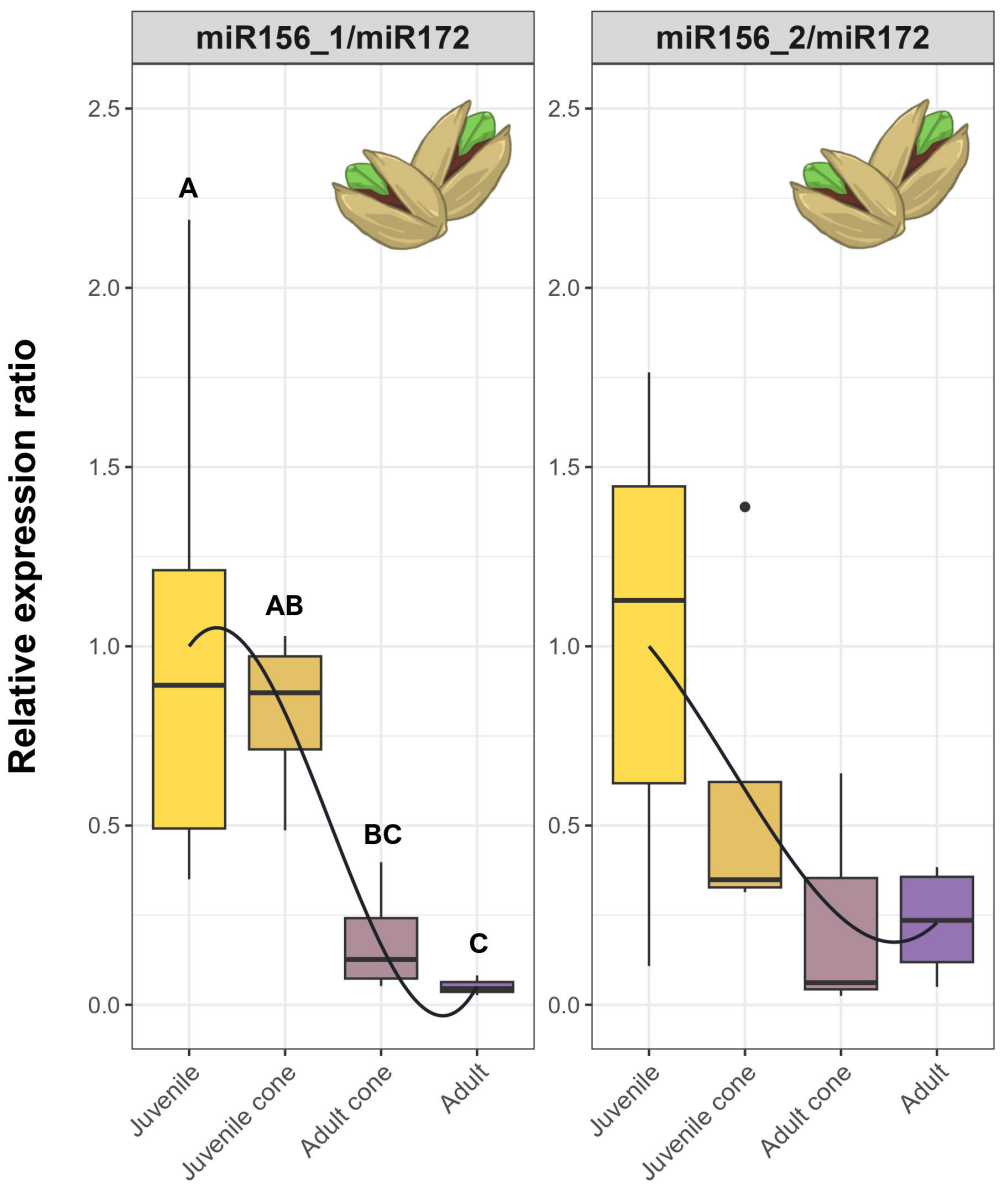
RT-qPCR results based on miRNA ratios across four tissue types of cultivated pistachio trees. Each plot represents RT-qPCR relative expression in four tissue types - Juvenile, Juvenile Cone, Adult Cone and Adult. Each box plot represents the range of values between Q1 (25%) and Q3 (75%). The black line in the boxplot represents the median (Q2). Dots outside the whiskers represent outliers. The curves were fitted to show trends, based on a polynomial function (see Methods). Significant differences between groups were calculated by Kruskal-Wallis test (P ≤ 0.05). The different groups, signified by letters above the boxplots, were determined using Dunn’s test (P ≤ 0.05).

Finally, spurred by the need to find practical markers for olive breeding, we investigated miRNAs and genes to determine which might be useful as selective markers. Genes like *AP2* and the *AGL42* homologs were promising, because they have distinct expression patterns in juvenile versus adult tissue (**Figure 4**). We also employed ratios of miRNA expression as markers, motivated by the fact that miRNA ratios are effective markers to predict and evaluate human disease (Avissar et al., 2009; Neely et al., 2010). A few ratios discriminated reliably between adult and juvenile tissues and exhibited gradual declines from seedlings to juvenile trees to juvenile cones to adults (**Figure 4**). If one assumes this developmental gradient is also related to the time to reproduction, miRNA ratios like miR156_1/miR172 and miR156_2/miR172 may be useful for selecting olive genotypes with short JPs. Further studies are needed to prove this point with at least two additional steps. First, the markers need to be applied to a large sample of trees that are followed from seedlings to reproductive maturity. With such data, one will be able to assess whether measured miRNA expression ratios in seedlings predict the time to heteroblasty and/or reproductive maturity. Second, even if the markers are useful for prediction in olives, they need to be assessed in other perennial crops. As an initial assessment, we assayed juvenile and adult cone tissues from pistachio (*Pistacia vera* L.) trees using the same primers and RT-qPCR methods applied to olive. We measured the ratios of miR156_1/miR172 and miR156_2/miR172 in leaf tissue from juvenile trees, adult trees and coned trees. Both ratios showed clear trends, with miR156_1/miR172 exhibiting a steady decrease from juvenile trees to juvenile cone tissue with statistical support for different groupings (**Figure 6**). It is worth noting that pistachios and olives are not closely related, since they represent different angiosperm orders. Thus, if these ratios are predictive for time to maturity, they may be applicable for a wide range of perennial crops and prove useful for marker-assisted selection of breeding materials with short juvenile periods.

## 4 Materials and methods

### 4.1 Plant material and sampling

The olive trees selected for this study were part of a breeding trial that included the evaluation of more than 1,000 F1 trees within the Olive Breeding Program of the University of Córdoba. The experimental field trial was located in Almensilla, Seville province, Spain (37° 18’ 51.1’’ N 6° 07’ 28.8’’ W). The trees were planted in 2015 in the same plot and managed with irrigation under the same agronomic conditions. In December 2018, we selected five trees with an evident juvenile cone. The five trees flowered for the first time in 2017 and bore fruits at the time of sampling. All five trees were full siblings derived from crossing ‘Koroneiki’ and ‘Arbosana’ cultivars (cv). Two additional trees from the same field that lacked a juvenile cone were included as controls for juvenile and adult stages. The juvenile control was a seedling from cv. Frantoio that was open-pollinated, and the adult control was cv. Arbosana.

All seven trees were sampled on the 4th of December 2018. To sample, we cut shoots that had sprouted in the previous year from each tree and then sampled fully expanded leaves located in the third and fourth nodes from the apex (Rubio-Valdés, 2009). In the five trees with juvenile cones, we sampled juvenile shoots from the bottom part of the cone (hereafter juvenile cone samples) and adult shoots from the upper part (hereafter adult cone samples) (**Supplementary Figure S1**). The adult shoots were bearing fruit, confirming their adult status. For the control plants, we sampled shoots from the entire canopy. A second sampling was carried out on the same seven trees the following year, in December of 2019.

Once we analyzed HTS data from the seven trees, we selected additional trees to verify the results. This new set included at least five olive trees per category, each aged 5 years but at different developmental stages: fully juvenile, fully adult, and olive seedlings (< 1 year old). The olive seedlings were germinated and grown in controlled conditions; these seedlings correspond to a deeper juvenile stage than even the 5-year-old juvenile trees. For every olive tree, we sampled leaves located in the third and fourth nodes of one-year shoots. To avoid complications due to seasonal variation in gene expression, we performed sampling in the same season (10 December 2019) as the previous year.

### 4.2 High throughput miRNA and RNA-seq data

Leaf samples were frozen in liquid nitrogen and stored at −80°C until RNA isolation. Total RNA was isolated from leaf material for all samples using the RNeasy® Plant Mini Kit (Qiagen – Germany) according to the manufacturer’s protocol. Frozen samples were ground to a fine powder in liquid nitrogen, and ~100 mg of tissue was used for RNA extraction. Additionally, the Qiagen RNase-Free DNase set was used for DNase treatment. Samples of isolated total RNA were stored at -80°C before use. The quantity and quality of the RNA samples were measured using the MaestroNano Micro-Volume Spectrophotometer (V-Bioscience) and the Bioanalyzer 2100 (Agilent Technologies), respectively.

To explore the expression of miRNAs, we constructed and sequenced a total of 16 small RNA libraries: 10 from the five trees with juvenile cones, which included two samples per tree from adult cone and juvenile cone leaves, an additional two biological replicates for both stages for one of the five trees, and one library each from the two controls, juvenile and adult. For each library, 1 μg of total RNA was fractionated on 15% denaturing polyacrylamide gel for size selection. The small RNAs (18 to 30 nts) were then ligated to Illumina TruSeq adapters at the 5’- and 3’-ends using the T4 RNA ligase. The ligated products were selected by size fractionation, and then adapter-ligated fractions were amplified for 15 cycles to produce sequencing libraries. The purified PCR products were sequenced with the HiSeq 2000 Sequencing System (Illumina, San Diego, CA, USA) according to the manufacturer’s protocol.

We also generated 16 RNA-seq libraries to investigate gene expression, using the same sampling scheme. RNA-seq libraries were prepared according to the manufacturer’s instructions (Kapa Stranded mRNA, Roche). Poly-A-containing mRNA was isolated from the total RNA, captured using magnetic oligo-dT beads, and fragmented using an RNA fragmentation kit. First-strand cDNA was generated using reverse transcriptase and random primers. Following the second strand cDNA synthesis and adaptor ligation, 200-bp cDNA fragments were isolated using gel electrophoresis and amplified by 15 cycles of PCR. The products were loaded onto an Illumina HiSeq4000 instrument and subjected to 75 cycles of paired-end (2 × 75 bp) sequencing. The processing of fluorescent images into sequences, base-calling and quality value calculations were performed using the Illumina data processing pipeline (version 1.8). RNA-seq and small RNA libraries were produced and sequenced at the Centre for Genomic Regulation (CRG, Spain).

### 4.3 Analysis of HTS data

#### 4.3.1 Small RNAs

Our sequencing resulted in a sequencing depth of ~50M reads per sample. The small RNA data was processed by removing adaptors, using Cutadapt (Martin, 2011), and filtering reads with insufficient quality (trimming nucleotides with a Phred quality score smaller than 20) or with length < 15 nucleotides using Sickle (Joshi and Fass, 2011). Reads were aligned to annotated non-coding transcripts of the Farga v8 olive reference genome (Oev8) (Cruz et al., 2016) using bwa (Li and Durbin, 2009); we pursued this approach to avoid studying possible mRNA fragments. We also discarded fragments that mapped to tRNA loci and noted all small RNAs that mapped to rRNA transcripts. To identify differentially expressed miRNA, we applied a methodology based on edgeR (Robinson et al., 2010) that measured the differential expression of each sequence in the juvenile and adult material based on count data for each unique miRNA. We applied upper quartile normalization to the raw counts and then estimated the common and tagwise dispersion. Then we performed an exact test based on a negative binomial law (Robinson and Smyth, 2008) and selected the sequences that showed differential expression with a false discovery rate (FDR) < 0.01. We performed edgeR analyses on two data sets: one that excluded small RNAs that mapped to rRNA loci and another that included those rRNA sequences. The inclusion of rRNA sequences increased sensitivity (see Results).

#### 4.3.2 RNA-seq

Each RNA-seq sample was sequenced to a depth of ~86M reads. The libraries were trimmed and filtered for quality using Trimmomatic (Bolger et al., 2014). We removed reads whose quality drops to a Phred score of 15 or below for 4 nucleotides and reads with length < 15. This reduced the yield to ~84M reads per sample on average. The remaining paired-end reads were aligned against the olive reference genome using a two-pass alignment strategy, which in principle improves the detection of splicing junctions (Veeneman et al., 2016), with the STAR program (Dobin et al., 2013). About 4.6% of the reads were deemed too short to be aligned, and only ~0.1% were unmapped for other reasons. Within the aligned reads, 82% were uniquely mapped to a single locus. The different transcripts in each sample were identified using StringTIE (Pertea et al., 2015). The identified loci were compared with the reference annotation of the genome using gffcompare (Pertea and Pertea, 2020), thus creating a merged annotation of the genome that represented a 38% increase in the number of identified loci with respect to the Oe8 annotation. The abundances of the different transcripts in each sample were measured using StringTIE, and the results were exported as a table to edgeR (Robinson et al., 2010) using the script prepDE.py3 from the StringTIE package. Counts were then normalized by the upper quartile, the tagwise and common dispersions were evaluated, and an exact test based on the negative binomial distribution was performed to determine differential expression. Only the loci with an FDR < 0.05 were considered significantly differentially expressed. Alternatively, abundances were computed based on the merged annotation using Cuffdiff (Trapnell et al., 2014) and the files with the processed reads for each case. Cuffdiff was run with standard parameters, using an FDR of 0.05 to test the significance of differential expression.

### 4.4 RT-qPCR validation

We validated expression patterns of differentially expressed miRNAs and genes by amplifying them in different years and sets of trees. These analyses used the RNA extraction methods described above and the same juvenile cone samples used for the small RNA and RNA-seq libraries. Independent RNA extractions were conducted for each analysis.

#### 4.4.1 Candidate and reference miRNAs

We performed RT-qPCR on candidate miRNAs and normalized the results against reference transcripts. No studies were identifying suitable miRNA references in olive, we analyzed our libraries to find miRNAs with high expression and low expression variability among tissues under our experimental conditions. To identify these references, we assumed that the statistical properties of the transcripts follow a negative binomial (NB) law (Robinson and Smyth, 2008), so that the expression variance is a function of the average expression across replicates multiplied by a sequence-dependent dispersion parameter. From the miRNA counts, we extracted fold change (FC) in expression for each miRNA between the juvenile and adult tissues, the average expression level of the adult sample, and the NB dispersion parameter. We then performed a multi-objective optimization to minimize the absolute value of log FC. Simultaneously, we minimized the NB dispersion parameter and maximized the average expression level. The resulting Pareto fronts were extracted using the R package rPref (Roocks, 2016). Finally, we selected the sequences that matched the expression level of the sample from the Pareto front and continued the procedure in an iterative fashion. For instance, if 5S rRNA was selected, it was removed and then the Pareto fronts were recomputed. Based on these procedures, we identified four short transcripts as references: ID_miR166-564, ID_miR166-825, snor21b, and 5S rRNA (see **Supplementary Table S3**).

miRNAs were amplified by RT-qPCR in a two-step reaction. Isolated RNA was retrotranscribed using the TaqMan MicroRNA Reverse Transcription kit (Applied Biosystems, Forster City, CA). For each sample (100 ng of total RNA), the reverse transcriptase (RT) step was multiplexed by including the stem-loop RT primers for miR156_1, miR156_2, miR159, and miR172, and the selected reference gene (5S rRNA). The synthesized cDNA was stored at -20°C. RT-qPCR reactions were carried out on LightCycler® 96 Instrument (Roche Life science) using the TaqMan™ Fast Advanced Master Mix (Applied Biosystems) following the manufacturer’s instructions. Prior to RT-qPCR, the cDNA was diluted 1:5, as established through optimization. Reactions were prepared in a total volume of 20 μl containing: 10 µl TaqMan Fast Advanced Master Mix (2x), 1 µl TaqMan Advanced miRNA Assay (20x), 7.67 µl nuclease-water and 1.33 µl cDNA template. The thermal-cycle profile of the qPCR started with 1 cycle of 20s at 95°C for polymerase activation, followed by 40 cycles of 3s at 95°C and 30s at 60°C. A no-template control (NTC) was included in each run to detect contamination. Primer amplification efficiencies were determined from a standard curve generated by serial dilutions (1, 1:4, 1:16, 1:64, 1:256) of cDNA for each miRNA and the reference gene (Bustin et al., 2009). The relative abundance of the four miRNA targets (miR156_1, miR156_2, miR159, and miR172) was determined using each miRNA efficiency curve. MIQE guidelines were followed during the whole experiment set-up and execution to ensure relevance, accuracy, correct interpretation, and repeatability of the assays that were being analyzed and compared (Bustin et al., 2009).

These assays required two sets of primers: for candidate miRNAs and potential references miRNAs. The formers were ordered from Thermo Scientific, based on the miRNA query sequence. The primer sequences are proprietary, so, similar to numerous previous papers on miRNA expression, we cannot provide the primer sequences (Reichel et al., 2015; Tang et al., 2012; Zheng et al., 2019). The reference miRNA primers were designed to capture the complete sequence of the non-coding transcript, based on the Oe8 annotations; miRNA sequences are reported in **Supplementary Table S4**.

#### 4.4.2 Candidate and reference genes

Gene expression was also validated by applying two-step RT-qPCR. Isolated RNA was retrotranscribed into cDNA using iScript cDNA synthesis (Bio-Rad) following the manufacturer’s instructions and then stored at -20°C. RT-qPCR was performed on the CFX Connect Real-Time PCR System (Bio-Rad) using the GoTaq qPCR Master Mix (Promega) according to the manufacturer’s instructions. The RT-qPCR program consisted of 1 cycle of 3 min at 95°C, 39 cycles of a) 15 s at 95°C and b) 30 s at 60°C, and 1 cycle of 3 min at 72°C, followed by a melting curve arranging from 60°C to 95°C, where 0.5°C were increased every 6 seconds. Primers (**Supplementary Table S5**) were designed using the NCBI primer designing tool: Primer-BLAST (Ye et al., 2012). All primer pairs were designed to ensure at least one intron gap, except for AP2_2 and AGL42_2, as this was not possible. Primer performance and amplification were checked by agarose gel and Sanger sequencing of the qPCR product.

To normalize qPCR results across samples, we identified five candidate genes based on the existing literature: Actin, Elongation Factor 1 alpha (EF1A), GAPDH, ubiquitin, and 18s (Alagna et al., 2016; Fernández-Ocaña et al., 2010; Gomez-Jimenez et al., 2010; Haberman et al., 2017; Ray and Johnson, 2014). Primers for these genes were taken from literature and are reported in **Supplementary Table S5**. To select those with the most stable expression regardless of cultivar and developmental stage, all five potential reference genes were measured by RT-qPCR following the previously described protocols in 20 trees at five different developmental stages: seedling (*n* = 5), juvenile (*n* = 5), juvenile cone (*n* = 5), adult cone (*n* = 5), and adult (*n* = 5). The resulting count values were evaluated using the software RefFinder (https://www.ciidirsinaloa.com.mx/RefFinder-master/) under manufacturer’s instructions.

### 4.5 Methylome generation and analysis

We generated BSseq data to investigate potential epigenetic differences between adult and juvenile tissue in plants with a juvenile cone. The data were generated for 6 samples representing three replicates of juvenile cone and adult cone materials from a single tree. BSseq was generated by the construction of the WGBS (Whole Genome sequencing of Bisulfite converted DNA) methyl-seq libraries, was carried out by the Centre for Genomic Regulation (CRG, Spain). We ran 6 libraries. Sequencing libraries were prepared according to the manufacturer’s instructions (Illumina, San Diego, CA). A total of 1.5 μg of genomic DNA was extracted from leaves and used to generate methyl-seq libraries. DNA was fragmented by sonication to 200-300bp with Covaris S220 sonicator, followed by end repair and adenylation. Then, the DNA fragments were treated with bisulfite using the EpiTect Bisulfite kit (Qiagen). The libraries were sequenced on a HiSeq 4000 system (Illumina) to obtain paired-end 100-bp reads per the manufacturer's instructions.

The resulting BSseq reads were trimmed to remove adapters and low-quality sequences using trimmomatic (v0.35) (Bolger et al., 2014) with default values. Reads were mapped to the Farga reference using Bismark (v0.15.0) (Krueger and Andrews, 2011) with bowtie2 (v 2.2.7) (Langmead and Salzberg, 2013) to align trimmed reads to the Oe8 reference genome, with seed parameters of -N 0 -L 20 and with post-bisulfite adapter tagging (--pbat). After alignment, we used the Bismark methylation extractor (0.15.0) to determine the numbers of bisulfite-converted and unconverted reads at each cytosine site.

We calculated the weighted methylation levels of predefined genomic windows and genome-wide levels using a custom R script (R version 4.0.2). Following (Schultz et al., 2012) the weighted methylation of any region was calculated separately for each cytosine context (CG, CHG, or CHH) as the number of methylation-supporting cytosine reads in that window divided by the number of unmethylated reads at cytosines in the same context. The methylation status of individual cytosines was determined with a one-sided binomial test against the bisulfite sequencing error rate. The error rate was calculated separately for each context by finding bisulfite conversion rates of unmethylated lambda phage DNA using Bismark. For these analyses, we considered the ranges of genes as all base pairs (bp) between their transcription start sites and transcription termination sites. Promoters were considered as the 2 kb region upstream (5’) of the transcription start site.

We identified differentially methylated regions (DMRs) using the methylKit package in R (v4.0.2) (Akalin et al., 2012) using the getMethylDiff() function with a q-value cutoff of ≤0.05 using SLIM correction and the logistic regression method. We classified three juvenile and three adult methylome samples as biological replicates of two different treatments. We required DMRs to have absolute methylation level difference cutoffs of 0.20, a minimum of 4 cytosines in the sequence context of interest, and a minimum of 4 reads per considered cytosine. Bins were 100 bp wide, and bins needed to be 200 bp apart to be considered separate DMRs. Due to the small number of DMRs identified with these criteria and fewer criteria, we considered all DMRs, including those that differed only in one context (e.g., only mCHH). We determined the overlap between DMRs and other feature types (genes, promoters, siRNA targets, miRNA, and repeats) via the IRanges and GenomicRanges packages in R (Aboyoun et al., 2013).

### 4.6 Additional statistical analyses

We evaluated the relative expression of miRNAs and genes between different developmental stages. For each miRNA, miRNA ratio, and gene, we compared their expression in juvenile and adult tissues with a Wilcoxon Rank test, using Statistix 10 Software. We used non-parametric Kruskal-Wallis tests to compare the relative expression of miRNAs, miRNA ratios, and genes among the five different developmental stages: seedling, juvenile, juvenile cone, adult cone, and adult. The test was implemented with the Kruskal-Wallis test module in R, both to determine statistical significance and to estimate η^2^, which quantifies the proportion of variance explained by the developmental stages. Dunn tests were applied after Kruskal-Wallis tests using Statistix 10 Software. The polynomial trend lines for expression across the five stages were fitted with the functions geom_smooth and poly(x, 3) included in the ggplot2 package following the method = "lm" in R.

## Data Availability

The data for this study have been deposited in the European Nucleotide Archive (ENA) at EMBL-EBI under accession number PRJEB100696 (https://www.ebi.ac.uk/ena/browser/view/PRJEB100696), Scripts used for data representation are available on GitHub (https://zenodo.org/records/14414732) (Romero-Rodríguez, 2024).
