# Supplementary material for: From juvenile to adult: investigating miRNAs, gene expression, and the juvenile cone in olive development
Source: Front Plant Sci. 2025 Oct 29;16:1682101. doi: 10.3389/fpls.2025.1682101 (PMC12605533; doi:10.3389/fpls.2025.1682101)
Supplement: Supplementary file 1 [file Table1.docx]

Supplementary Material

**Table S1:** List of the DEGs, their coordinates on the reference genome (OE6) and p-values

| **gene_id** | **OE6 Gene** | **p_value (CuffDiff)** | **q_value** | **p-value (EdgeR)** | **FDR** |
| --- | --- | --- | --- | --- | --- |
| MSTRG.8499 | OE6A058044 | 0.0002 | 0.0489515 |  |  |
| MSTRG.3515 | OE6A069835 | 0.0002 | 0.0489515 |  |  |
| MSTRG.22989 | OE6A018260 | 0.00015 | 0.0408197 |  |  |
| MSTRG.22200 | OE6A094910 | 0.00015 | 0.0408197 |  |  |
| MSTRG.16968 | OE6A044379 | 5.00E-05 | 0.0198865 |  |  |
| MSTRG.64740 | OE6A061670 | 5.00E-05 | 0.0198865 |  |  |
| MSTRG.43085 | OE6A044902 | 5.00E-05 | 0.0198865 |  |  |
| MSTRG.20499 | OE6A022683 | 5.00E-05 | 0.0198865 |  |  |
| MSTRG.29073 | OE6A069646 | 5.00E-05 | 0.0198865 |  |  |
| MSTRG.52790 | OE6A035069 | 5.00E-05 | 0.0198865 |  |  |
| MSTRG.17418 | OE6A015727, OE6A038162 | 0.0001 | 0.0313364 |  |  |
| MSTRG.8174 | OE6A107347 | 5.00E-05 | 0.0198865 |  |  |
| MSTRG.57292 | OE6A053508 | 5.00E-05 | 0.0198865 |  |  |
| MSTRG.17690 | OE6A012806 | 5.00E-05 | 0.0198865 |  |  |
| MSTRG.36585 | OE6A092841 | 5.00E-05 | 0.0198865 |  |  |
| MSTRG.4526 | OE6A049811 | 5.00E-05 | 0.0198865 |  |  |
| MSTRG.9219 | OE6A063960 | 0.0001 | 0.0313364 |  |  |
| MSTRG.39673 | OE6A043442, OE6A080650 | 5.00E-05 | 0.0198865 |  |  |
| MSTRG.22411 | OE6A084474 | 5.00E-05 | 0.0198865 |  |  |
| MSTRG.11728 | OE6A105042 | 5.00E-05 | 0.0198865 |  |  |
| MSTRG.29586 | OE6A035993 | 5.00E-05 | 0.0198865 |  |  |
| MSTRG.15459 | OE6A057397 | 5.00E-05 | 0.0198865 |  |  |
| MSTRG.52970 | OE6A107315 | 5.00E-05 | 0.0198865 |  |  |
| MSTRG.66994 | OE6A037378 | 0.0001 | 0.0313364 |  |  |
| MSTRG.43830 | OE6A110363 | 5.00E-05 | 0.0198865 |  |  |
| MSTRG.61919 | OE6A103433 | 5.00E-05 | 0.0198865 |  |  |
| MSTRG.39859 | OE6A079697 | 0.0001 | 0.0313364 |  |  |
| MSTRG.61587 | OE6A104962 | 0.00015 | 0.0408197 |  |  |
| MSTRG.60535 | OE6A063302 | 5.00E-05 | 0.0198865 |  |  |
| MSTRG.18935 | OE6A090898 | 5.00E-05 | 0.0198865 |  |  |
| MSTRG.27483 | OE6A031752 | 5.00E-05 | 0.0198865 |  |  |
| MSTRG.52525 | OE6A076402 | 5.00E-05 | 0.0198865 |  |  |
| MSTRG.48162 | OE6A041977 | 5.00E-05 | 0.0198865 |  |  |
| MSTRG.38278 | OE6A076012 | 5.00E-05 | 0.0198865 |  |  |
| MSTRG.58160 | - | 0.00015 | 0.0408197 |  |  |
| MSTRG.37995 | OE6A037275 | 0.0002 | 0.0489515 |  |  |
| MSTRG.68606 | OE6A098446 | 0.00015 | 0.0408197 |  |  |
| MSTRG.49813 | OE6A002405 | 0.00015 | 0.0408197 |  |  |
| MSTRG.15742 | OE6A006876 | 0.0001 | 0.0313364 |  |  |
| MSTRG.26839 | OE6A110943 | 5.00E-05 | 0.0198865 |  |  |
| MSTRG.27829 | OE6A018608 | 5.00E-05 | 0.0198865 |  |  |
| MSTRG.46382 | OE6A029275 | 5.00E-05 | 0.0198865 |  |  |
| MSTRG.45306 | OE6A110555 | 5.00E-05 | 0.0198865 |  |  |
| MSTRG.16583 | OE6A096920 | 5.00E-05 | 0.0198865 |  |  |
| MSTRG.27830 | - | 0.0002 | 0.0489515 |  |  |
| MSTRG.8946 | OE6A040680 | 5.00E-05 | 0.0198865 |  |  |
| MSTRG.8865 | OE6A119490 | 0.00015 | 0.0408197 |  |  |
| MSTRG.55049 | OE6A017217 | 0.0002 | 0.0489515 |  |  |
| MSTRG.2918 | OE6A000389 | 0.0002 | 0.0489515 |  |  |
| MSTRG.28253 | OE6A003512 | 0.0002 | 0.0489515 |  |  |
| MSTRG.9254 | OE6A079850 | 0.00015 | 0.0408197 |  |  |
| MSTRG.56422 | OE6A103785 | 0.0002 | 0.0489515 |  |  |
| MSTRG.57344 | OE6A115706 | 5.00E-05 | 0.0198865 |  |  |
| MSTRG.20906 | OE6A024472 | 5.00E-05 | 0.0198865 |  |  |
| MSTRG.51760 | - | 0.0002 | 0.0489515 |  |  |
| MSTRG.23666 | OE6A092579 | 5.00E-05 | 0.0198865 |  |  |
| MSTRG.53141 | OE6A059151 | 5.00E-05 | 0.0198865 |  |  |
| MSTRG.66281 | OE6A068401 | 5.00E-05 | 0.0198865 |  |  |
| MSTRG.59052 | OE6A009091 | 5.00E-05 | 0.0198865 |  |  |
| MSTRG.66791 | OE6A081277 | 0.00015 | 0.0408197 |  |  |
| MSTRG.45335 | OE6A021400 | 0.0001 | 0.0313364 |  |  |
| MSTRG.19910 | OE6A075985 | 5.00E-05 | 0.0198865 |  |  |
| MSTRG.43840 | - | 0.0001 | 0.0313364 |  |  |
| MSTRG.4419 | OE6A069005 | 0.00015 | 0.0408197 |  |  |
| MSTRG.32521 | OE6A110393 | 0.0001 | 0.0313364 |  |  |
| MSTRG.30691 | OE6A085795 | 5.00E-05 | 0.0198865 |  |  |
| MSTRG.35953 | OE6A012609 | 0.00015 | 0.0408197 |  |  |
| MSTRG.5457 | OE6A037807 | 0.0001 | 0.0313364 |  |  |
| MSTRG.11453 | OE6A040429 | 5.00E-05 | 0.0198865 |  |  |
| MSTRG.40453 | OE6A079551 | 0.0001 | 0.0313364 |  |  |
| MSTRG.18166 | OE6A025827 | 0.00015 | 0.0408197 |  |  |
| MSTRG.29954 | OE6A109618 | 0.0002 | 0.0489515 |  |  |
| MSTRG.51073 | OE6A040088 | 0.00015 | 0.0408197 |  |  |
| MSTRG.55654 | OE6A116409 | 0.00015 | 0.0408197 |  |  |
| MSTRG.14412 | OE6A020794, OE6A069553, OE6A083237, OE6A115664 | 0.0001 | 0.0313364 |  |  |
| MSTRG.12636 | OE6A106493 | 5.00E-05 | 0.0198865 |  |  |
| MSTRG.8588 | OE6A113527 | 0.0001 | 0.0313364 |  |  |
| MSTRG.11490 | OE6A034465 | 5.00E-05 | 0.0198865 |  |  |
| MSTRG.58111 | OE6A050167 | 5.00E-05 | 0.0198865 |  |  |
| MSTRG.44301 | OE6A075652 | 5.00E-05 | 0.0198865 |  |  |
| MSTRG.7124 | OE6A053599 | 5.00E-05 | 0.0198865 |  |  |
| MSTRG.26700 | OE6A101780 | 0.00015 | 0.0408197 |  |  |
| MSTRG.688 | OE6A026338 | 5.00E-05 | 0.0198865 |  |  |
| MSTRG.43674 | OE6A007424, OE6A064538 | 5.00E-05 | 0.0198865 |  |  |
| MSTRG.3289 | OE6A053514 | 5.00E-05 | 0.0198865 |  |  |
| MSTRG.10478 | OE6A050487 | 5.00E-05 | 0.0198865 |  |  |
| MSTRG.50531 | OE6A103454 | 5.00E-05 | 0.0198865 |  |  |
| MSTRG.31196 | OE6A092227 | 5.00E-05 | 0.0198865 |  |  |
| MSTRG.54973 | OE6A073508 | 0.0002 | 0.0489515 |  |  |
| MSTRG.55530 | OE6A013920 | 5.00E-05 | 0.0198865 |  |  |
| MSTRG.68631 | OE6A080376 | 5.00E-05 | 0.0198865 |  |  |
| MSTRG.41962 | OE6A063786 | 0.00015 | 0.0408197 |  |  |
| MSTRG.41913 | OE6A069433 | 0.0002 | 0.0489515 |  |  |
| MSTRG.19619 | OE6A031759 | 5.00E-05 | 0.0198865 |  |  |
| MSTRG.47435 | OE6A037993 | 0.0002 | 0.0489515 |  |  |
| MSTRG.29989 | OE6A077746 | 5.00E-05 | 0.0198865 |  |  |
| MSTRG.40227 | OE6A091982 | 5.00E-05 | 0.0198865 |  |  |
| MSTRG.24928 | OE6A097413 | 5.00E-05 | 0.0198865 |  |  |
| MSTRG.45455 | OE6A027275 | 0.0001 | 0.0313364 |  |  |
| MSTRG.37864 | OE6A113763 | 5.00E-05 | 0.0198865 |  |  |
| MSTRG.49142 | OE6A100587 | 0.0001 | 0.0313364 |  |  |
| MSTRG.48048 | OE6A099504 | 5.00E-05 | 0.0198865 |  |  |
| MSTRG.25108 | OE6A071229 | 0.0001 | 0.0313364 |  |  |
| MSTRG.25598 | OE6A116144 | 5.00E-05 | 0.0198865 |  |  |
| MSTRG.13455 | - | 5.00E-05 | 0.0198865 |  |  |
| MSTRG.42870 | OE6A115531 | 0.00015 | 0.0408197 |  |  |
| MSTRG.19370 | OE6A083347 | 5.00E-05 | 0.0198865 |  |  |
| MSTRG.3723 | OE6A109227 | 5.00E-05 | 0.0198865 |  |  |
| MSTRG.16249 | OE6A024135 | 5.00E-05 | 0.0198865 |  |  |
| MSTRG.18682 | OE6A047738 | 5.00E-05 | 0.0198865 |  |  |
| MSTRG.56569 | OE6A047591 | 0.0001 | 0.0313364 |  |  |
| MSTRG.6648 | OE6A115487 | 0.0002 | 0.0489515 |  |  |
| MSTRG.63063 | OE6A081379 | 5.00E-05 | 0.0198865 |  |  |
| MSTRG.23791 | - | 0.00015 | 0.0408197 |  |  |
| MSTRG.49660 | OE6A088521 | 5.00E-05 | 0.0198865 |  |  |
| MSTRG.37658 | OE6A090531 | 5.00E-05 | 0.0198865 |  |  |
| MSTRG.54654 | OE6A104718 | 0.0001 | 0.0313364 |  |  |
| MSTRG.17974 | OE6A005599 | 5.00E-05 | 0.0198865 |  |  |
| MSTRG.29379 | OE6A067978 | 5.00E-05 | 0.0198865 |  |  |
| MSTRG.38724 | OE6A056993 | 5.00E-05 | 0.0198865 |  |  |
| MSTRG.4736 | OE6A090615 | 5.00E-05 | 0.0198865 |  |  |
| MSTRG.30631 | OE6A004112 | 5.00E-05 | 0.0198865 |  |  |
| MSTRG.20425 | OE6A013675 | 5.00E-05 | 0.0198865 |  |  |
| MSTRG.22132 | OE6A012339 | 5.00E-05 | 0.0198865 |  |  |
| MSTRG.45512 | OE6A036345 | 5.00E-05 | 0.0198865 |  |  |
| MSTRG.61657 | OE6A074644 | 5.00E-05 | 0.0198865 |  |  |
| MSTRG.42324 | OE6A008390 | 5.00E-05 | 0.0198865 |  |  |
| MSTRG.22638 | OE6A048146 | 5.00E-05 | 0.0198865 |  |  |
| MSTRG.1048 | - | 0.0001 | 0.0313364 |  |  |
| MSTRG.10345 | OE6A000683 | 5.00E-05 | 0.0198865 |  |  |
| MSTRG.60834 | OE6A060161 | 5.00E-05 | 0.0198865 |  |  |
| MSTRG.39634 | OE6A094307 | 0.0001 | 0.0313364 |  |  |
| MSTRG.56408 | OE6A022364 | 0.0001 | 0.0313364 |  |  |
| MSTRG.49423 | OE6A042612 | 0.0001 | 0.0313364 |  |  |
| MSTRG.69316 | - | 5.00E-05 | 0.0198865 |  |  |
| MSTRG.49779 | OE6A059352 | 5.00E-05 | 0.0198865 |  |  |
| MSTRG.67733 | OE6A003441 | 5.00E-05 | 0.0198865 |  |  |
| MSTRG.34117 | OE6A118246 | 5.00E-05 | 0.0198865 |  |  |
| MSTRG.27392 | OE6A021146, OE6A044381 | 5.00E-05 | 0.0198865 |  |  |
| MSTRG.54333 | OE6A036682 | 5.00E-05 | 0.0198865 |  |  |
| MSTRG.61376 | OE6A050105 | 5.00E-05 | 0.0198865 |  |  |
| MSTRG.44160 | OE6A092418 | 5.00E-05 | 0.0198865 | 1.68E-07 | 0.00271416 |
| MSTRG.64114 | OE6A101307 | 0.0001 | 0.0313364 |  |  |
| MSTRG.20624 | OE6A034712 | 5.00E-05 | 0.0198865 |  |  |
| MSTRG.37104 | OE6A002088 | 0.0001 | 0.0313364 |  |  |
| MSTRG.31777 | OE6A072380 | 0.00015 | 0.0408197 |  |  |
| MSTRG.8967 | OE6A071261 | 5.00E-05 | 0.0198865 |  |  |
| MSTRG.3446 | OE6A091892 | 5.00E-05 | 0.0198865 |  |  |
| MSTRG.30023 | OE6A051225 | 0.0001 | 0.0313364 |  |  |
| MSTRG.21177 | OE6A115651 | 0.0002 | 0.0489515 |  |  |
| MSTRG.65064 | OE6A057634 | 5.00E-05 | 0.0198865 |  |  |
| MSTRG.42417 | OE6A101649 | 0.0001 | 0.0313364 |  |  |
| MSTRG.28396 | OE6A008998 | 5.00E-05 | 0.0198865 |  |  |
| MSTRG.28894 | OE6A018517, OE6A027274, OE6A045550, OE6A068407 | 5.00E-05 | 0.0198865 |  |  |
| MSTRG.31248 | OE6A035881 | 5.00E-05 | 0.0198865 |  |  |
| MSTRG.46231 | OE6A001028 | 0.0001 | 0.0313364 |  |  |
| MSTRG.58982 | OE6A004824 | 0.0001 | 0.0313364 | 1.97E-07 | 0.00271416 |
| MSTRG.20356 | OE6A035977 | 5.00E-05 | 0.0198865 |  |  |
| MSTRG.4471 | OE6A033137 | 5.00E-05 | 0.0198865 | 9.50E-06 | 0.04094986 |
| MSTRG.8362 | OE6A103168 | 0.0001 | 0.0313364 |  |  |
| MSTRG.63040 | OE6A015620 | 5.00E-05 | 0.0198865 |  |  |
| MSTRG.44998 | OE6A064763 | 0.0002 | 0.0489515 |  |  |
| MSTRG.37489 | OE6A115278 | 0.00015 | 0.0408197 |  |  |
| MSTRG.18015 | OE6A015535 | 5.00E-05 | 0.0198865 |  |  |
| MSTRG.68433 | OE6A018218 | 5.00E-05 | 0.0198865 |  |  |
| MSTRG.1256 | OE6A071497 | 5.00E-05 | 0.0198865 |  |  |
| MSTRG.52265 | OE6A058749 | 5.00E-05 | 0.0198865 |  |  |
| MSTRG.29280 | OE6A019660 | 5.00E-05 | 0.0198865 | 2.37E-12 | 8.19E-08 |
| MSTRG.33893 | - | 0.0002 | 0.0489515 |  |  |
| MSTRG.13081 | OE6A111924 |  |  | 4.31E-81 | 2.97E-76 |
| MSTRG.8165 | OE6A004694 |  |  | 2.79E-06 | 0.02137319 |
| MSTRG.29278 | OE6A026361 |  |  | 7.90E-09 | 0.00018165 |
| MSTRG.40520 | OE6A010654 |  |  | 9.30E-06 | 0.04094986 |
| MSTRG.10588 | OE6A066749 |  |  | 8.44E-07 | 0.0083184 |
| MSTRG.42062 | OE6A075379 |  |  | 1.16E-05 | 0.04699654 |
| MSTRG.33753 | OE6A116692 |  |  | 6.92E-06 | 0.03981163 |
| MSTRG.24921 | OE6A104526 |  |  | 6.89E-07 | 0.00791964 |
| MSTRG.34576 | OE6A099815 |  |  | 8.70E-06 | 0.04094986 |
| MSTRG.45127 | OE6A073929 |  |  | 1.31E-06 | 0.01128364 |
| MSTRG.20406 | OE6A060525 |  |  | 3.80E-06 | 0.02620744 |
| MSTRG.12591 | OE6A013032 |  |  | 7.96E-06 | 0.04094986 |
| MSTRG.60486 | OE6A054547 |  |  | 5.29E-06 | 0.03319802 |
