# Supplementary material for: From juvenile to adult: investigating miRNAs, gene expression, and the juvenile cone in olive development
Source: Front Plant Sci. 2025 Oct 29;16:1682101. doi: 10.3389/fpls.2025.1682101 (PMC12605533; doi:10.3389/fpls.2025.1682101)
Supplement: Supplementary file 2 [file Table2.docx]

Supplementary Material

**Table S2:** Sample size (*n*) of each evaluated group.

| **Category** | **Seedling** | **Juvenile** | **Juvenile cone** | **Adult cone** | **Adult** |
| --- | --- | --- | --- | --- | --- |
| small RNAs and RNAseq HTS libraries | - | 1 | 5^1^ | 5^1^ | 1 |
| RT-qPCR validation on miRNAs | 8 | 6 | 5 | 5 | 8 |
| RT-qPCR validation on genes^2^ | 7 | 10 | 5 | 5 | 10 |

1. We performed two biological replicates for one of the selected, i.e., seven libraries were constructed and sequenced for each tissue type of juvenile cone: juvenile and adult.

2. The expression of *AP2_1* was measured in groups of five individuals (*n =* 5).
