# Supplementary material for: From juvenile to adult: investigating miRNAs, gene expression, and the juvenile cone in olive development
Source: Front Plant Sci. 2025 Oct 29;16:1682101. doi: 10.3389/fpls.2025.1682101 (PMC12605533; doi:10.3389/fpls.2025.1682101)
Supplement: Supplementary file 3 [file Table3.docx]

Supplementary Material

**Table S3:** Results of Kruskal-Wallis test on expression data, as measured across five developmental stages, based on miRNA ratios and candidate genes.

| **Category** | **Measure** | ***p*-value** | **η^2^** |
| --- | --- | --- | --- |
| **miRNA ratio** | miR156_1/miR172 | 0.001083 | 0.52924 |
|  | miR156_2/miR172 | 0.0003652 | 0.61805 |
|  | miR159/miR172 | 8.448e-05 | 0.73624 |
|  | miR156_1/miR159 | 0.000393 | 0.61209 |
| **Genes** | AGL42_1 | 0.0001763 | 0.57123 |
|  | AGL42_2 | 0.0003583 | 0.52280 |
|  | AP2_1 | 0.0003242 | 0.52964 |
|  | AP2_2 | 6.042e-06 | 0.79845 |
|  | CAL | 0.0008871 | 0.46038 |
