# Supplementary material for: From juvenile to adult: investigating miRNAs, gene expression, and the juvenile cone in olive development
Source: Front Plant Sci. 2025 Oct 29;16:1682101. doi: 10.3389/fpls.2025.1682101 (PMC12605533; doi:10.3389/fpls.2025.1682101)
Supplement: Supplementary file 4 [file Table4.docx]

Supplementary Material

**Table S4:** Sequence information for the potential miRNA reference loci.

| **Candidate reference gene** | **Class** | **Mature sequence-5p (5’- 3’)** |
| --- | --- | --- |
| ID_miR166-564 | MicroRNA | CGGACCAGGCUUCAUUCCCU |
| ID_miR166-825 | MicroRNA | UCGGACCAGGCUUCGUUCCUU |
| snor21b | Small nucleolar RNA | GGGUGCGACCAUACCAGCACUAACGCACCGGAUCCCAUCAGAACUCCGAAGUUAAGCGUGCUUGGGCGAGAGUAGUACUAGGAUGGGUGACCCCCUGGGAAGUCCUCGUGUUGCACCCC |
| 5s rRNA | Ribosomal RNA | GAGCUGUGAUGAGAAAUUGUCAUGCACCACUCUGACUAUUAUCAGGUUGAUGAUAAUUUUAUGUACCCAUUCAAUUUCUGAGCUC |
