# Supplementary material for: From juvenile to adult: investigating miRNAs, gene expression, and the juvenile cone in olive development
Source: Front Plant Sci. 2025 Oct 29;16:1682101. doi: 10.3389/fpls.2025.1682101 (PMC12605533; doi:10.3389/fpls.2025.1682101)
Supplement: Supplementary file 5 [file Table5.docx]

Supplementary Material

**Table S5:** The candidate genes and the assayed reference genes. It provides gene ID plus the primer sequences. For the primer’s taken from the literature, the correct reference is cited.

| **Name** | **Sequence (5'-3')** | **Bibliography** | **OE6 Gene ID** |
| --- | --- | --- | --- |
| Actin-A | AAGATCAAAGTTGTTGCACCACC | Haberman *et al.* 2017 | - |
| Actin-B | CTTAGAAATCCACATCTGCTGGAAT |  |  |
| EF1A-F | CTGACTGCGCCGTCCTTATC | Alagna *et al.* 2016 | - |
| EF1A-R | TGACACCAAGGGTGAAGGC |  |  |
| GAPDH-F | ACAGCTCCTGGTAAGGGTGA | Ray and Johnson 2014 | - |
| GAPDH-R | GGCTTGCGTCAAGAAGTCTC |  |  |
| Ubiquitin-1 | ATGCAGATCTTTGTGAAGAC | Gomez-Jimenez *et al.* 2010 | - |
| Ubiquitin-2 | ACCACCACGAAGACGGAG |  |  |
| 18s-F | GTGACGGGTGACGGAGAATT | Fernández-Ocaña *et* *al.* 2010 | - |
| 18s-R | GACACTAATGCGCCCGGTAT |  |  |
| ABS-F | CGACGCTGTTGGTGTTTATGG | - | OE6A069433 |
| ABS-R | AATCTTGTGCCTGTGGGCAT |  |  |
| AGL42_1-F | AGTTCAACAACATGTGCAGCC | - | OE6A063960 |
| AGL42_1-R | GTCCCCAAGTCTTGCCCTAAA |  |  |
| AGL42_2-F | CAAGCAGGTTGGTTACCTTCTC | - | OE6A063960 |
| AGL42_2-R | TGGAGCTTGAGAACTGAAAAAGTC |  |  |
| AP2_1-A | CCAGTTGATGTCCCAACCTGA | - | OE6A019660 |
| AP2_1-B | ATCACTCTGGACAAAGTGCTGG |  |  |
| AP2_2-F | TAACATCGCTTGGTGCGAGT | - | OE6A026361 |
| AP2_2-R | AAGACGGTTCCTATGCGTCC |  |  |
| CAL-F | GATTTGGGTCCCCTCAGCTT | - | OE6A018260 |
| CAL-R | AGCTCGGAAATTGACTCGTC |  |  |
| UNK-F | TCACGCCATAGTGCGAATGT | - | OE6A058044 |
| UNK-R | GAGGCTTTGGGTGTCGGATT |  |  |
