# Supplementary material for: From juvenile to adult: investigating miRNAs, gene expression, and the juvenile cone in olive development
Source: Front Plant Sci. 2025 Oct 29;16:1682101. doi: 10.3389/fpls.2025.1682101 (PMC12605533; doi:10.3389/fpls.2025.1682101)
Supplement: Supplementary file 6 [file Image1.pdf]

Supplementary Material

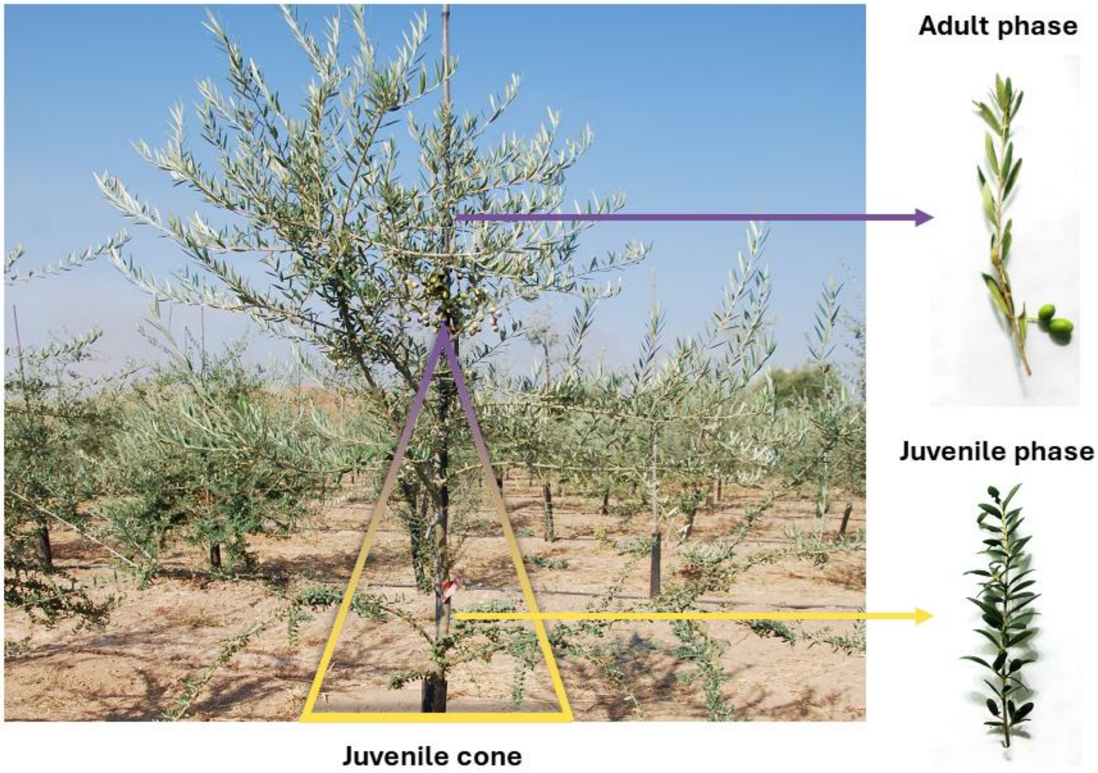

Figure S1: Olive in juvenile cone developmental stage. The upper and peripheral parts of the plants consist of adult tissue, while the basal and inner parts remain juvenile.
