# Supplementary material for: From juvenile to adult: investigating miRNAs, gene expression, and the juvenile cone in olive development
Source: Front Plant Sci. 2025 Oct 29;16:1682101. doi: 10.3389/fpls.2025.1682101 (PMC12605533; doi:10.3389/fpls.2025.1682101)
Supplement: Supplementary file 7 [file Image2.pdf]

frontiers

Supplementary Material

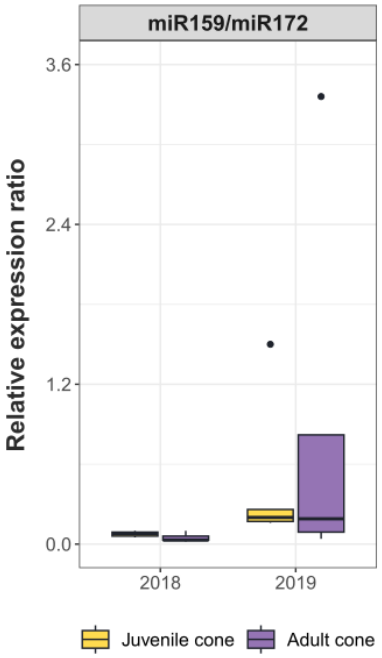

Figure S2: Boxplots of the one remaining miRNA relative expression ratio not included in Figure 4A. Each box plot represents the range of values between Q1 (25%) and Q3 (75%). The black line in the boxplot represents the median (Q2). Dots outside the whiskers represent outliers. As in Figure 4A, relative expression for adult and juvenile tissue was measured for two years in a sample of five plants with juvenile cones.
