# Supplementary material for: From juvenile to adult: investigating miRNAs, gene expression, and the juvenile cone in olive development
Source: Front Plant Sci. 2025 Oct 29;16:1682101. doi: 10.3389/fpls.2025.1682101 (PMC12605533; doi:10.3389/fpls.2025.1682101)
Supplement: Supplementary file 8 [file Image3.pdf]

Supplementary Material

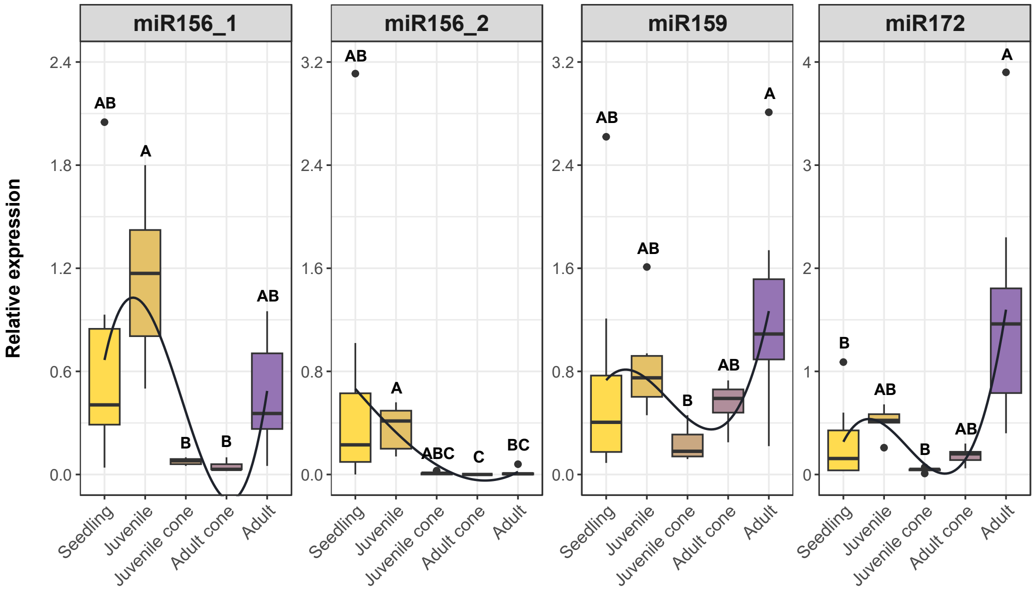

Figure S3: Expression relative to a control for individual candidate miRNAs in five distinct tissue stages. Each box plot represents the range of values between Q1 (25%) and Q3 (75%). The black line in the boxplot represents the median (Q2). Dots outside the whiskers represent outliers. The curves were fitted to show trends, based on a polynomial function (see Methods). Significant differences between groups were calculated by Kruskal-Wallis test ($P < 0.05$). The different groups, signified by letters above the boxplots, were determined using Dunn's test ($P < 0.05$).
