# Supplementary material for: From juvenile to adult: investigating miRNAs, gene expression, and the juvenile cone in olive development
Source: Front Plant Sci. 2025 Oct 29;16:1682101. doi: 10.3389/fpls.2025.1682101 (PMC12605533; doi:10.3389/fpls.2025.1682101)
Supplement: Supplementary file 9 [file Image4.pdf]

Supplementary Material

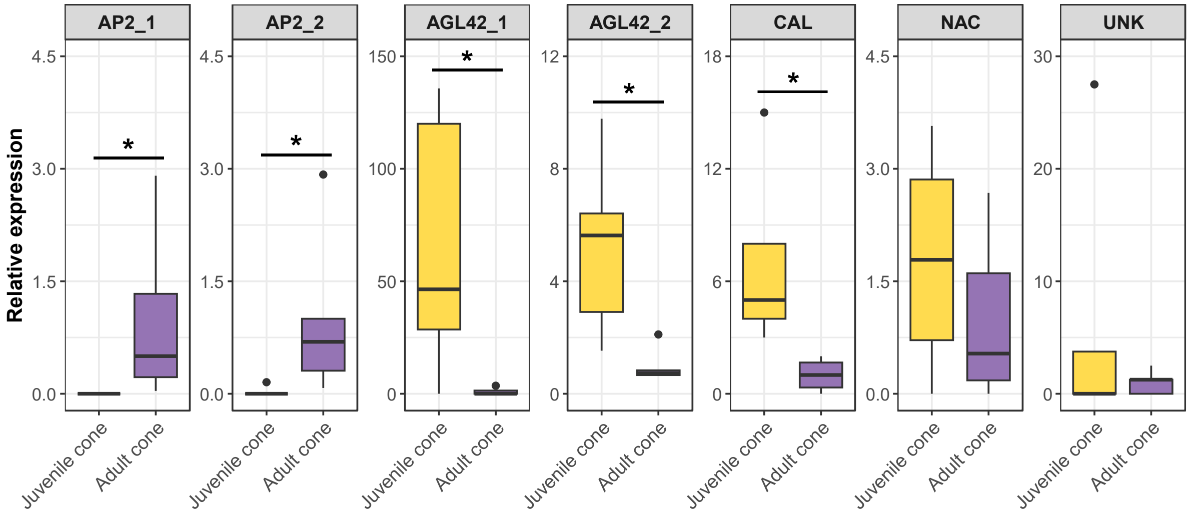

Figure S4: Relative expression of candidate genes based on RT-qPCR assays. Each candidate gene was assayed in juvenile cone and adult cone tissue from five trees. Each box plot represents the range of values between Q1 (25%) and Q3 (75%). The black line in the boxplot represents the median (Q2). Dots outside the whiskers represent outliers. Significant differences between groups were calculated by Kruskal-Wallis test; significant differences ($P < 0.05$) denoted by an asterisk.
