# Supplementary material for: From juvenile to adult: investigating miRNAs, gene expression, and the juvenile cone in olive development
Source: Front Plant Sci. 2025 Oct 29;16:1682101. doi: 10.3389/fpls.2025.1682101 (PMC12605533; doi:10.3389/fpls.2025.1682101)
Supplement: Supplementary file 10 [file Image5.pdf]

Supplementary Material

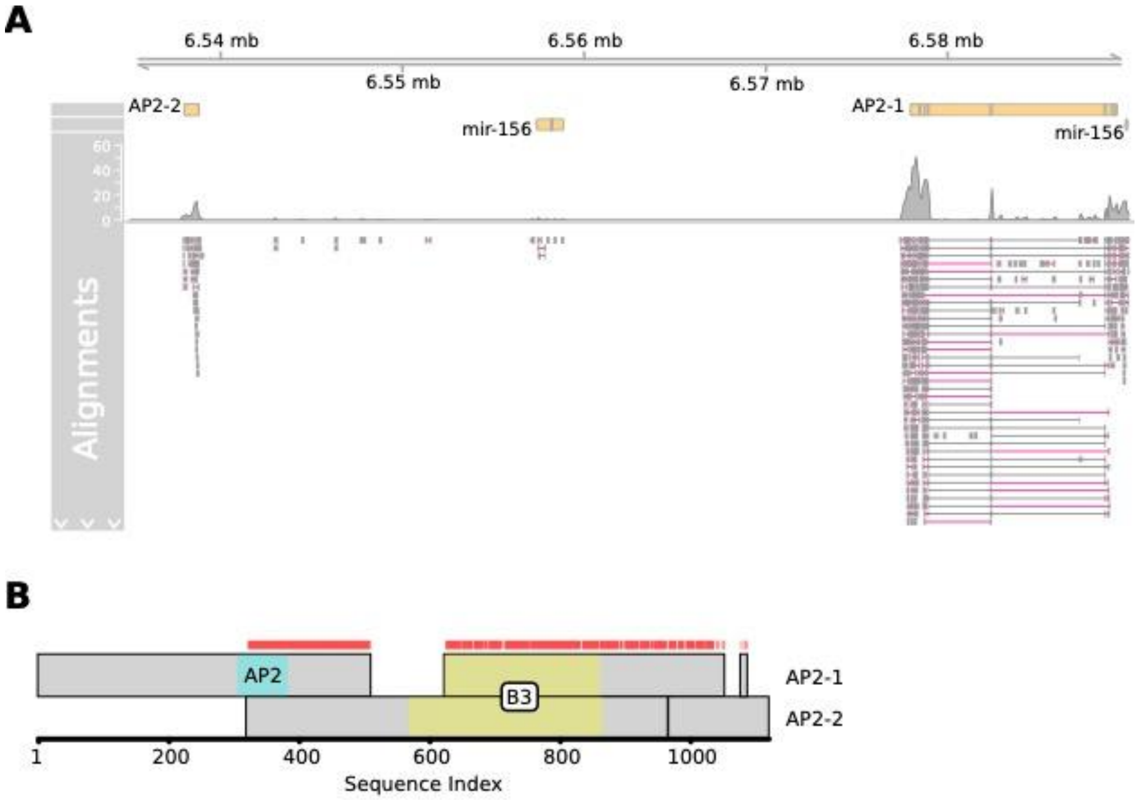

Figure S5: A. A diagram indicating the genomic organization of AP2_1, AP2_2 and miR156 genes, all of which are located in a 400 kb region. Note the alternance of miR156 and the AP2 genes. B. Alignment diagram of AP2_1 and AP2_2. The red line indicates the identity of the nucleotides. Also indicated are the regions identified functionally as AP2 and B3 by BLASTp.
