# Supplementary material for: From juvenile to adult: investigating miRNAs, gene expression, and the juvenile cone in olive development
Source: Front Plant Sci. 2025 Oct 29;16:1682101. doi: 10.3389/fpls.2025.1682101 (PMC12605533; doi:10.3389/fpls.2025.1682101)
Supplement: Supplementary file 12 [file Image7.pdf]

Supplementary Material

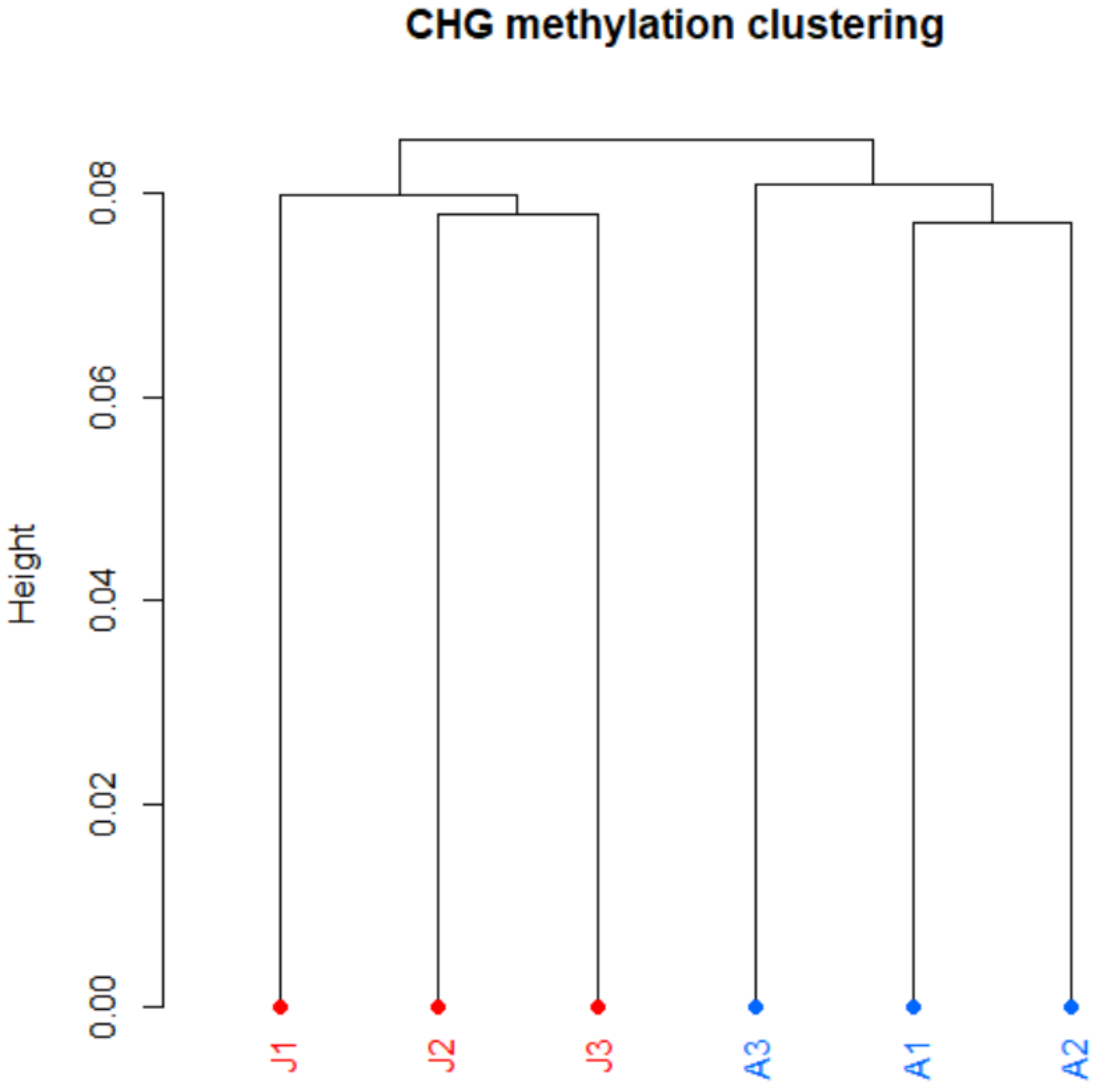

Figure S7: Clustering of samples based on whole genome methylome data. The figure is analogous to figure 5B, except for the CHH context.
