# Supplementary material for: From juvenile to adult: investigating miRNAs, gene expression, and the juvenile cone in olive development
Source: Front Plant Sci. 2025 Oct 29;16:1682101. doi: 10.3389/fpls.2025.1682101 (PMC12605533; doi:10.3389/fpls.2025.1682101)
Supplement: Supplementary file 13 [file Image8.pdf]

Supplementary Material

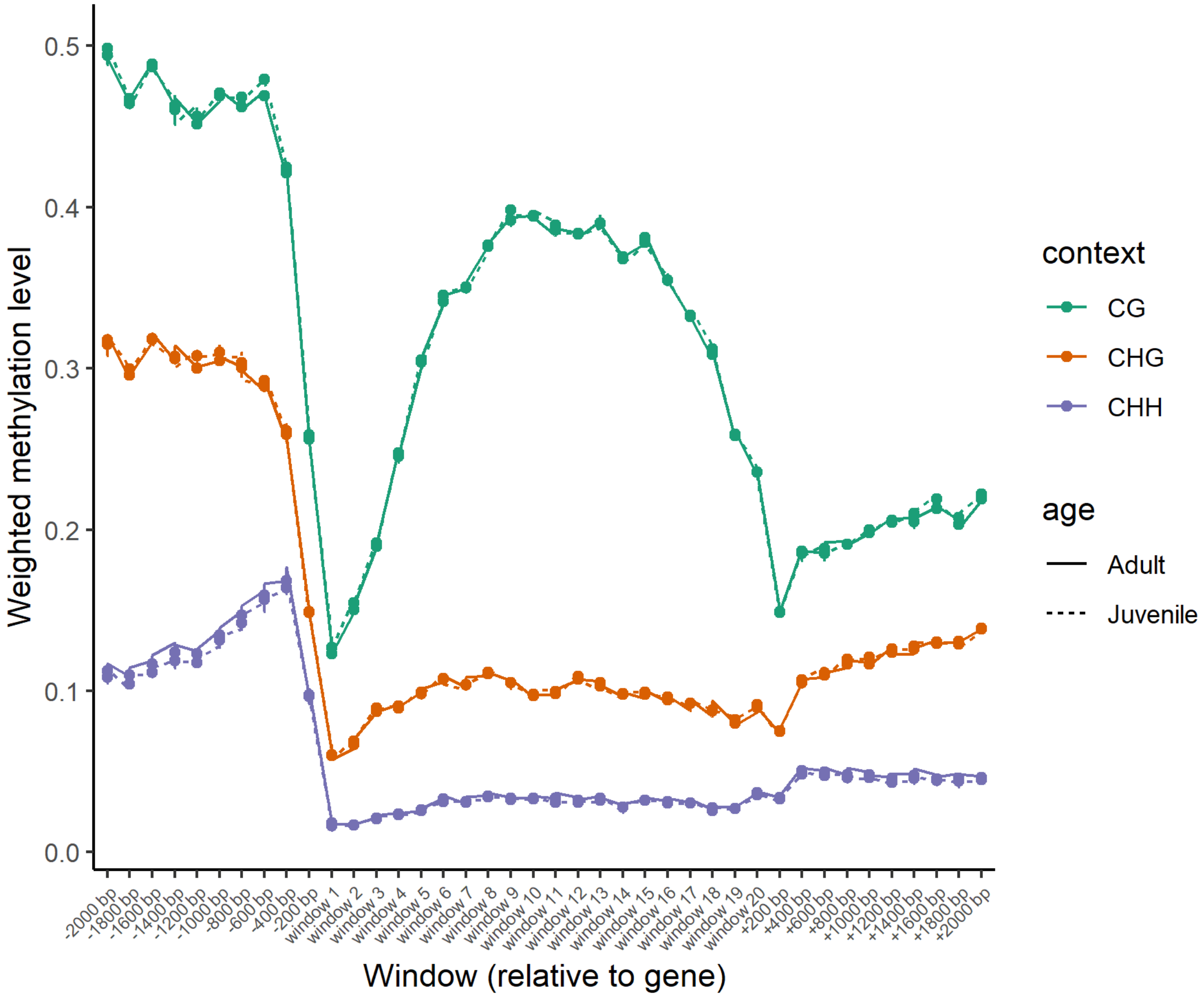

Figure S8: Profiles of methylation across genes and their 2.0 kb 5′ and 3′ flanking regions. Weighted methylation levels are summarized in 10 200 bp windows upstream and downstream of genes, and in 20 equally sized windows within genes that vary in size depending on gene length. These plots are virtually identical for adult and juvenile tissue.
